# Genetic Variants in Preeclampsia: Lessons From Studies in Latin-American Populations

**DOI:** 10.3389/fphys.2018.01771

**Published:** 2018-12-14

**Authors:** Rafael Tomoya Michita, Valéria de Lima Kaminski, José Artur Bogo Chies

**Affiliations:** Immunogenetics Laboratory, Department of Genetics, Biosciences Institute, Universidade Federal do Rio Grande do Sul, Porto Alegre, Brazil

**Keywords:** preeclampsia, vasculopathy, endothelial damage, inflammation, SNPs, Latin America, polymorphism

## Abstract

Placental vascularization is a tightly regulated physiological process in which the maternal immune system plays a fundamental role. Vascularization of the maternal-placental interface involves a wide range of mechanisms primarily orchestrated by the fetal extravillous trophoblast and maternal immune cells. In a healthy pregnancy, an immune cross-talk between the mother and fetal cells results in the secretion of immunomodulatory mediators, apoptosis of specific cells, cellular differentiation/proliferation, angiogenesis, and vasculogenesis, altogether favoring a suitable microenvironment for the developing embryo. In the context of vasculopathy underlying common pregnancy disorders, it is believed that inefficient invasion of extravillous trophoblast cells in the endometrium leads to a poor placental blood supply, which, in turn, leads to decreased secretion of angiogenic factors, hypoxia, and inflammation commonly associated with preterm delivery, intrauterine growth restriction, and preeclampsia. In this review, we will focus on studies published by Latin American research groups, providing an extensive review of the role of genetic variants from candidate genes involved in a broad spectrum of biological processes underlying the pathophysiology of preeclampsia. In addition, we will discuss how these studies contribute to fill gaps in the current understanding of preeclampsia. Finally, we discuss some trending topics from important fields associated with pregnancy vascular disorders (e.g., epigenetics, transplantation biology, and non-coding RNAs) and underscore their possible implications in the pathophysiology of preeclampsia. As a result, these efforts are expected to give an overview of the extent of scientific research produced in Latin America and encourage multicentric collaborations by highlighted regional research groups involved in preeclampsia investigation.

## Introduction

In all pregnancies that can potentially lead to living birth, a major concern is the high prevalence of disorders that can affect healthy pregnancies. Maternal mortality is a global health issue. One of the eight goals of the United Nations Millennium Development Goals (MDG) was to reduce maternal mortality by three quarters from 1990 to 2015. As of 2013, the worldwide maternal mortality ratio has dropped 45%, yet maternal deaths are still the primary cause of death. For the same period, an estimated 289,000 maternal deaths due to pregnancy- or childbirth-related complications occurred, particularly in developing countries, since mortality rates vary according to geographical area and different social and ethnic characteristics. These estimates expose the alarming healthcare situation in developing countries where the maternal mortality ratio is ~14 times higher than in developed countries. Actual numbers might be even higher because only 51% of the countries evaluated in the MDG had data on maternal causes of death (United Nations, 2015). In Latin America, pregnancy vascular disorders are the leading cause of maternal mortality and morbidity (Khan et al., 2006). These disorders cover a wide range of clinically characterized phenotypes with a common underlying dysfunction in the endothelial and vascular systems, including preeclampsia (PE), and will be appropriately discussed in this review.

Owing to a lack of robust experimental animal models and ethical issues related to early pregnancy tissue usage, elucidation of the underlying mechanisms involved in the pathophysiology of pregnancy disorders remains the “holy grail” of reproductive biology. Considering that fetal cells inherit half paternal genetic material, this “non-self” status (compared to the mother) represents a challenge to the maternal immunological system. In this sense, a question naturally arises: How does the fetus avoid rejection by the maternal immune system? Since rejection occurs at different levels, it is reasonable to consider that genetic disparity, or the genetic background of the parents may account for an increased risk of pregnancy disorders (Goldenberg et al., 2009; Gardosi et al., 2013; Lisonkova and Joseph, 2013). Human pregnancy is a phenomenon that relies on immunological adaptations (Aghaeepour et al., 2017). Since maternal immune tolerance is essential to the maintenance of pregnancy, breakage of such tolerance is an accepted hypothesis for the occurrence of pregnancy-related disorders, including PE (Christiansen, 2013; Redman et al., 2014), which is briefly reviewed in sections Placental Vasculogenesis and Angiogenesis: Immune System and Vascular Remodeling During Pregnancy and Preeclampsia.

Pregnancy is a highly coordinated process that requires the involvement of a well-regulated network of biological mechanisms. Briefly, pregnancy establishment initiates through blastocyst implantation and endometrial invasion. Blastocyst invasion requires the expression of a wide range of factors by both maternal and fetal cells, including adhesion molecules, pregnancy hormones, and inflammatory mediators (Norwitz et al., 2001). In this context, inefficient blastocyst implantation is related to impaired endometrial vascular remodeling and immunological tolerance, which are commonly observed in a broad spectrum of pregnancy disorders. The extent of maternal physiological responses driven by the foreign developing embryo involves both maternal/paternal and fetal aspects. The response for such stimuli varies between healthy and pathological pregnancies, or even among individuals of the same group. This implies that the genetic variability is a critical component and accounts in the susceptibility for (but not limited to) pregnancy vascular disorders by influencing both local and systemic responses. In Latin America, the genetic and molecular basis of PE is a rapidly developing field of investigation, and many studies approaching basic science or even extending to cutting-edge technologies have been published and will be reviewed in the sections Genetic Studies in Latin-American Populations, Genetic Variation in Histocompatibility-Related Genes in PE, Gene Variants Involved in Metabolic Processes, and Variants in Detoxification, DNA-Repair, and Apoptosis-Related Genes.

**Figure 1:**
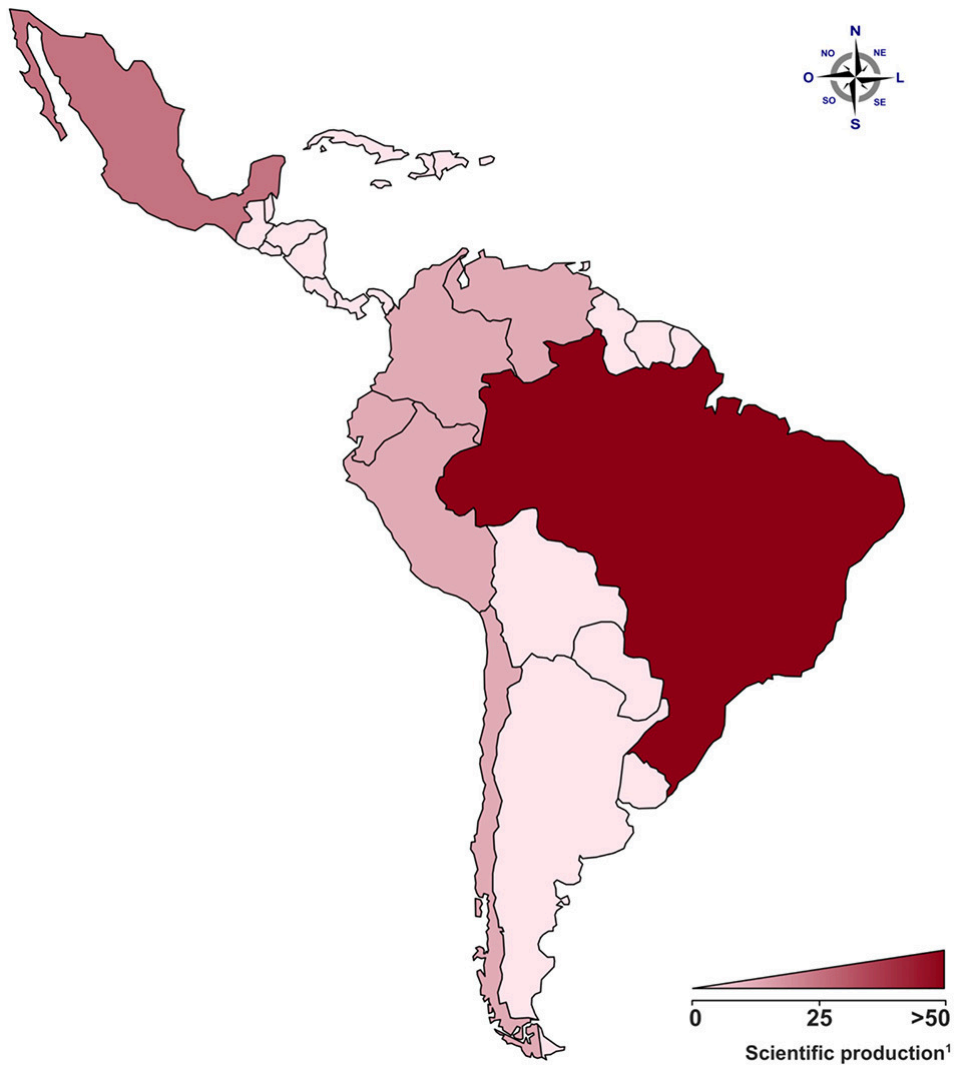
The contribution from Latin American countries to the understanding of genetic predisposition in pregnancy vascular disorders. In the map, areas in red represent the number of published articles covered by our review in Latin America. Areas in weak red represent low numbers of publications, and areas in strong red represent countries/populations with a production of 50 articles or more.

Latin America contains a highly diverse human population. This admixed population is also under the influence of environmental factors, such as climate, lifestyle, and pathogen exposure. As pregnancy disorders are affected by both genetic and environmental factors, it is difficult to extrapolate data obtained in specific human populations to other ones. Therefore, we provide an extensive review of studies developed in Latin America (Figure 1) as a contribution to the understanding of pregnancy disorders, mainly focusing on PE. Since Brazil and Mexico are at the forefront of PE investigation in Latin America, we call attention to the lack of investigative studies in countries not represented here. Also, we highlight the urgent need for collaborative studies and extensive efforts to fill gaps in the current scenario of hypertensive pregnancy disorder epidemiology in Latin America. Here, we will discuss current knowledge about the role of the maternal immune system in pregnancy vasculogenesis and PE. Also, we will review the literature concerning genetic studies evaluating the contribution of single nucleotide polymorphisms (SNPs) in candidate genes from distinct biological systems and discuss their involvement in PE pathogenesis by analyzing data from Latin America as well as from other human populations when appropriate. For the sake of clarity, reference SNP cluster (rs#) will be cited as it appears in the text and the SNP nomenclature will be maintained according to the original cited article.

## Placental Vasculogenesis and Angiogenesis: Immune System and Vascular Remodeling During Pregnancy

Tissue remodeling and angiogenesis are the results of a tightly regulated interaction between the immune system and the vascular system (Ribatti and Crivellato, 2009). In pregnancy, an adequate placental vascularization depends on the proliferation and differentiation of the trophoblast cells in the placental villi (Herr et al., 2010). Adaptation and changes in maternal anatomy and physiology are fundamental for the establishment of an adequate blood supply for the developing fetus (Boeldt and Bird, 2017). After implantation, the invasion of the endometrium by the cytotrophoblast drives the first steps of human placentation. Initially, myometrial spiral arteries are remodeled in the second trimester, changing from a high-resistance state of coiled vessels to dilated low-resistance vessels (Boeldt and Bird, 2017). In low-resistance vessels, the exchange of gas and nutrients is highly facilitated, since there is a decrease in blood flow to the intervillous spaces of the placenta (Boeldt and Bird, 2017). According to the immunological aspects of pregnancy, it is accepted that a mild pro-inflammatory stimulus is essential for local tissue remodeling, neovascularization, and the establishment of successful embryo attachment enabling fetal development (Chaouat, 2002). Decidual immune cells, invading trophoblasts and endothelial cells interact and orchestrate placental vascularization. Leukocytes represent 15–30% of all cells in human early pregnant decidua (Mincheva-Nilsson et al., 1994). The organization of these immune cells is unique and includes lymphoid cell clusters, and randomly distributed immune cells, such as uterine natural killer (uNK) cells, αβ-T, and γδ-T cells, dendritic cells (DCs), and macrophages. B cells and regulatory B cells are less represented in number, and their emerging roles in pregnancy are discussed elsewhere (Muzzio et al., 2013; Fettke et al., 2014; Mor et al., 2017; Esteve-Solé et al., 2018). uNK cells represent ~70% of leukocytes in the decidua (Moffett-King, 2002), and are essential to the angiogenesis and maintenance of vascular stability by secreting specific sets of cytokines: the vascular endothelial growth factor C (VEGFC), the placental growth factor (PIGF), and angiopoietin 2 (ANG2) (Li et al., 2001).

## Preeclampsia

Worldwide, PE affects 2–8% of pregnant women. In addition, it accounts for ~40% of preterm births (< 35 weeks of gestation) (Khan et al., 2006; Duley, 2009). PE incidence differs mainly between low- and high-income countries. In Latin American countries, ~26% of maternal deaths are attributed to PE. However, the actual impact of PE in developing countries is underestimated due to differences in PE diagnostic criteria and the fact that reporting the maternal cause of death is not compulsory in several countries (Giachini et al., 2017).

PE usually manifests in the second trimester. Although new definitions for PE include organ dysfunction (Tranquilli et al., 2014) and no longer require proteinuria if other severe PE features are present (ACOG, 2013), traditionally PE is defined by onset of hypertension after 20 weeks of gestation (systolic ≥140 mmHg; diastolic ≥90 mmHg), proteinuria (≥300 mg/24 h or protein/creatinine ratio ≥0.5 in random sample) and edema. While untreated PE can be lethal, the clinical complications vary and include seizures, liver rupture, pulmonary edema, and renal insufficiency (Adu-Bonsaffoh et al., 2013). Despite advances in the clinical management of PE (symptomatic treatment), the only effective treatment remains clinical intervention and delivery, resulting in low birth weight and premature birth. In fact, ~23% of low birth weight and ~20% of preterm birth occurrences in Latin America are attributed to PE (Bilano et al., 2014). In clinical practice, therapies involving antiplatelet agents such as low aspirin doses (Duley et al., 2007; Roberge et al., 2013; Xu et al., 2015; ACOG, 2018) and calcium supplementation in women with low calcium diets (Hofmeyr et al., 2014) have proven to bring small to moderate benefit to women with high risk pregnancies. Symptomatic treatments include different strategies targeting gestational hypertension (antihypertensive therapy), eclamptic seizures (anticonvulsive therapy), and other symptoms as reviewed elsewhere (Ramos et al., 2017).

The impact of PE on both maternal and fetal health goes beyond pregnancy, and represents a significant burden on public health services, especially, in low-income countries where the incidence rates can reach up to 6% in Latin America, 2.3% in Africa, and 3.2% in Asia (Bilano et al., 2014). Preeclamptic women have an increased risk of post-partum depression, cardiovascular disorders, metabolic diseases and hypertension later in life (Ramsay et al., 2003; Hoedjes et al., 2011; Behrens et al., 2017; Neiger, 2017; Timpka et al., 2017; Zoet et al., 2018), while newborns are at higher risk to develop autistic spectrum disorders, cerebral palsy, and bronchopulmonary dysplasia due to low birth weight and preterm birth (Hansen et al., 2010; Mann et al., 2010; Strand et al., 2013).

**Figure 2:**
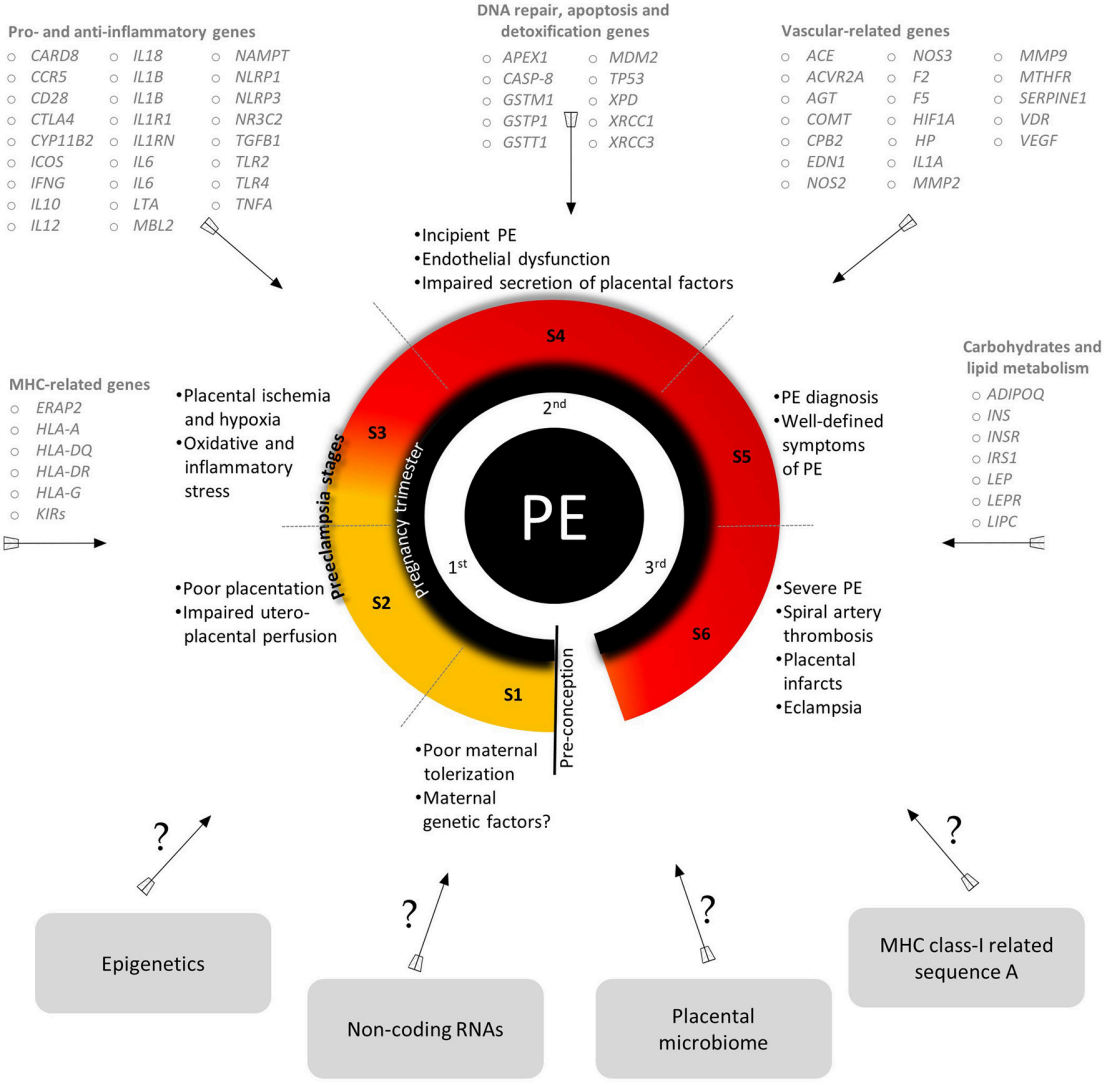
An integrated view of the genes evaluated by Latin American research groups, a summary of key events of PE stages and areas of future investigation in PE. S, stages. Genes are listed according to their approved gene symbol by the HUGO gene nomenclature committee. Detailed information about PE stages are found in Redman et al. (2014). All the genes mentioned and their SNPs are addressed in detail in Tables 1–5.

Despite extensive efforts in the last two decades, the etiopathology of PE is still unclear, although some environmental and genetic risk factors have been reported (Fong et al., 2014; Ye et al., 2017). The variety of candidate genes evaluated by Latin American research groups and the critical events of each stage of PE development are summarized in Figure 2 (for more details see Redman, 2014; Redman et al., 2014). Classically, PE development follows a two-stage model including a pre-clinical and a clinical period (Redman, 1991). This model was recently updated into a sequential four- and six-stage model to accommodate all immune aspects of PE: In the first stage of PE, environmental and genetic factors represent a critical component. The latter element involves several genes from different signaling pathways, revealing the polygenic nature of PE (for example, it is suggested that limited exposure to paternal antigens likely increases PE risk, being clinically relevant in primiparous women). In the next stage, inefficient trophoblast invasion in the decidua may result in poor placentation and abnormal uteroplacental perfusion. In the third stage, placental ischemia and hypoxia result in local oxidative stress and inflammatory response. Secondary to placental damage, in the fourth stage, impaired secretion of placental and maternal factors lead to the manifestation of the clinical symptoms of PE. In the fifth stage, diagnosis of PE is clear. At this stage, the vascular damage is augmented in response to systemic inflammation (i.e., Th1/Th17 cytokines). The last stage characterizes a more severe form of the disorder (observed in up to 40% of placentas) and involves atherosis, a focal lesion in the spiral arterial wall associated with placental infarction and arterial thrombosis (Harsem et al., 2007).

Placental hypoxia and impaired perfusion lead to the release of reactive oxygen species (ROS) and endothelial damage. Thus, the release of fetal cell debris and syncytiotrophoblast microparticles into maternal circulation prompts an intense pro-inflammatory response by maternal immune cells (Redman and Sargent, 2000; Sibai et al., 2005). Also worth mentioning is the pregnancy stress test hypothesis, which postulates that pregnancy is a maternal stress test for the vascular, metabolic or immunological systems (Williams, 2003; Roberts and Hubel, 2010; Myatt and Roberts, 2015). Following this idea, women with pre-existing vascular dysfunction would present a lower threshold for the stress test, and a higher predisposition to develop PE and chronic disorders later in life.

PE might also be the manifestation of two extreme situations converging in a common phenotype. Sometimes, in maternal PE, normal placentation occurs in women with the pre-existing chronic disease. Conversely, in placental PE, abnormal placentation results in poor placental perfusion (Valenzuela et al., 2012). This concept highlights a not exclusive dependency of PE in placentation failure and explains the variability of clinical phenotypes and timing of PE development.

Familial history and hypertensive disorders increase the risk of PE, implying that the genetic components are also risk-modifying factors (Bezerra et al., 2010). PE is a polygenic disorder, and although no single genetic variant is believed to be responsible for all cases of PE, individual *loci*, environmental factors, and epistasis are components that should not be neglected (Staines-Urias et al., 2012; Williams, 2016). In this sense, the evaluation of genetic variants in PE risk could partially explain disorder susceptibility and would be of great importance to identify candidate targets for gene-gene interaction analyses, as well as to better follow-up/management of women at higher risk.

## Genetic Studies in Latin-American Populations

### Pro- and Anti-inflammatory Mediators in PE

In Latin America, several immune-related genes have been evaluated, and most of the studies are summarized in Table 1. For example, costimulatory molecules play a role in immune cell differentiation and activation, SNPs in the *CTLA4* (rs231775), *CD28* (rs3116496), and *ICOS* (rs4675378) were evaluated in Brazilian women with PE (Pendeloski et al., 2011). An association between the *ICOS* (−1564 T/C) SNP and PE was suggested based on a lower frequency of the *ICOS* “T” allele and the “TT” genotype in PE cases compared to controls. A systemic inflammatory response mediated by cytokines can cause endothelial damage, and thus it plays a central role in PE severity. In this scenario, six SNPs of pro-inflammatory genes were studied: *IL1R1* (rs2234650), *IL12* (rs3212227), *IL18* (rs187238), *IL18* (rs1946519), *TLR2* (rs5743708), and *TLR4* (rs4986790). However, no differences in genotypic and allelic frequencies between PE and controls were observed (Franchim et al., 2011). In a Northern Mexico population study, the association between PE risk and the *TGFB1* SNPs: −800G/A (rs1800468), −509C/T (rs1800469), and +869T/C (rs1800470) and their haplotypes were evaluated. No association between PE development and the SNPs or haplotypes was observed, although the +869TT genotype was suggested as a protective factor against severe PE (Aguilar-Duran et al., 2014).

**Table 1:** Summary of studies developed in Latin America evaluating the role of genetic variation in pro- and anti-inflammatory mediators in PE.

**Factors**	**Sample size^†^**	**Key findings**	**Country**	**References**
*ICOS* (T-1564C) *CTLA4* (A49G) *CD28* (T17C)	130/260	Association with protection for PE: ICOS−1564T allele and−1564TT genotype.	Brazil	Pendeloski et al., 2011
*TGFB1* (G800A, C509T, T869C)	175/253	Association with protection for severe PE: TGFB1 869TT genotype.	Mexico	Aguilar-Duran et al., 2014
*IL1R1* (rs2234650) *IL12* (rs3212227) *IL18* (rs187238, rs1946519) *TLR2* (rs5743708) *TLR4* (rs4986790)	109/174	No association with PE.	Brazil	Franchim et al., 2011
*TNFA* (G308A) *IL6* (G174C) *IFNG* (A874T) *IL10* (A1082G, C819T, C592A) *TGFB1* (T869C, G915C)	165/101^a^	No association with PE.	Brazil	de Lima et al., 2009
*TNFA* (G308A) *TGFB1* (T10C, C25G) *IL10* (G1082A) *IL6* (G174C) *IFNG* (A874T)	151/189^b^	Association with PE risk: IL10−1082GG genotype in white women.	Brazil	Daher et al., 2006
*IL1B* (rs1143630)	169/287	Association with PE risk: IL1B rs1143630 ‘T' allele.	Brazil	Leme Galvão et al., 2016
*TNFA* (G308A,C850T)	105/200	No association with PE.	Mexico	Canto-Cetina et al., 2007
*IL10* (G1082A) *IL6* (G174C) *IL1RA* (86bp-VNTR)	411/613	No association with PE.	Mexico	Valencia Villalvazo et al., 2012
*TNFA* (G308A) *IL6* (G-174C) *IFNG* (A874T) *IL10* (A1082G, C819T, C592A) *TGFB1* (T869C,G915C)	116/165^c^	Association with protection for PE: IL6−174C allele.	Brazil	Pinheiro et al., 2015
*MBL2* allele B (rs1800450), allele C (rs1800451), allele D (rs5030737)	157/162	Association with PE severity: “AD” genotype, “C” and “D” alleles.	Brazil	Vianna et al., 2010
*CCR5* (CCR5Δ32)	155/144	Association with protection for PE: CCR5Δ32 allele.	Brazil	Telini et al., 2014
*NAMPT* (rs3801266)	389/212^d^	Association with GH: rs3801266 “AG” and “GG” genotypes.	Brazil	Luizon et al., 2015
*NAMPT* (rs1319501; rs3801266)	379/207^e^	Association with PE risk: rs1319501 “TC+CC” and rs3801266 “AG+GG” genotypes.	Brazil	Luizon et al., 2017
*LTA* (+252A>G)	30/115	No association with PE.	Brazil	Pissetti et al., 2015
*NLRP1* (rs11651270, rs12150550, rs2670660) *NLRP3* (rs35829419, rs10754558) *CARD8* (rs2043211, rs6509365) *IL1B* (rs1143634)	286/309	Association with risk for PE: rs12150220 (L155H) and the “rs11651270/C-rs12150220/A-rs2670660/A” haplotype.	Brazil	Pontillo et al., 2015
*CYP11B2* (T344C) *MR* (S810L)	100/100	No association with PE.	Mexico	Ramírez-Salazar et al., 2011
*CYP11B2* (T344C)	185/118^f^	No association with PE.	Brazil	de Vasconcelos et al., 2009

† Pooled cases/controls.

a Cases were grouped according severity: PE (n = 92) and eclampsia (n = 73).

b Studied population was grouped according to skin color (white and non-white); white: PE (n = 56) and control (n = 92); non-white: PE (n = 95) and control (n = 97).

c Cases were compared to healthy pregnant (n = 107) and non-pregnant women (n = 58).

d Cases correspond to PE (n = 208) and gestational hypertension (GH) cases (n = 181).

e Cases were grouped according to disorder severity and response to anti-hypertensive therapy: PE responsive (n = 60) and non-responsive (n = 145); GH responsive (n = 120) and non-responsive (n = 54).

f *Cases were grouped in PE (n = 70) and GH (n = 115)*.

Since different cytokine profiles have been associated with PE development (Saito and Sakai, 2003), de Lima et al. (2009) investigated SNPs of cytokine genes in eclampsia and PE in northwestern Brazilian individuals. They evaluated the SNPs *TNFA* (−308 G>A), *IL6* (−174 G>C), *IFNG* (+874 A>T), *IL10* (−1082 A>G, −819 C>T, −592 C>A), and *TGFB1* (+869 T>C, +915 G>C). No differences in genotypes and allelic frequencies were observed (de Lima et al., 2009). However, in a previous study by the same group, individuals were stratified according to ethnic origin in Caucasian and non-Caucasian, and the association of PE with *TNFA* (−308), *TGFB1* (+10;25), *IL10* (−1082), *IL6* (−174), and *IFNG* (+874) SNPs was evaluated. Intriguingly, the *IL10* −1082G/G SNP was associated with PE in Caucasian women, which is the most frequent allelic variant in people of African ancestry (Daher et al., 2006). A possible association between SNPs in *IL1B* was investigated in Brazilian women with severe PE. In this study, the “rs1143630 T” allele was associated with PE (Leme Galvão et al., 2016). In a Maya-Mestizo population sample, no association between *TNFA* (−308G/A, −850C/T) SNPs and PE was observed (Canto-Cetina et al., 2007). Another study reported no association of *IL10* (rs1800896), *IL6* (rs1800795), and *IL1RA* variable number of tandem repeats (VNTR) in intron 2 with PE susceptibility in Mexican-Mestizo women and Maya-Amerindian women from Mexico (Valencia Villalvazo et al., 2012).

The influence of *TNFA, IL6, IFNG*, and *IL10* gene SNPs and their relationship with cytokine plasma levels in severe PE, normotensive pregnancy, and in non-pregnant women from Brazil was investigated by Pinheiro et al. (2015). The SNPs evaluated in the study were *TNFA* (−308), *TGFB1* (+10;25), *IL10* (−1082), *IL6* (−174), and *IFNG* (+874). Interesting, they observed higher IL-10 levels in normotensive pregnant women compared to preeclamptic women. Conversely, higher plasma levels of IL-6 and IFN-γ were detected in PE in comparison to non-pregnant and normotensive pregnant women. Also, a positive correlation between IFN-γ plasma levels and the *IFNG* +874T allele was observed, and when the three groups were evaluated separately, a positive correlation between IL-6 levels and the presence of the IL6 −174C allele in normotensive pregnant women was observed (Pinheiro et al., 2015).

Mannose-binding lectin (MBL) is a pro-inflammatory protein that modulates inflammation and ultimately induces apoptosis (Turner, 2003). Polymorphisms in the *MBL2* gene located at exon 1: at codons 54 (allele B, rs1800450), 57 (allele C, rs1800451), and 52 (allele D, rs5030737) were evaluated in women with PE and in healthy pregnant controls from Brazil. The absence of all the variants characterize the wild-type allele “A.” In this study, an association between genotypes coding for low MBL levels and a severe PE was evidenced. In the AD genotype, the C and D alleles were more frequent in PE compared to controls. Moreover, in relation to MBL levels, three groups of haplotypes were observed: group 1 (H/L)YA/(H/L)YA and (H/L)YA/LXA genotypes were related to high MBL serum levels; group 2 encompasses LXA/LXA and (H/L)YA/O genotypes, which were related to intermediate MBL serum levels; and group 3 was defined by low MBL serum levels, resulting in MBL deficiency, corresponding to LXA/O and O/O genotypes (Vianna et al., 2010). Cysteine-cysteine chemokine receptor type 5 (CCR5) is an essential receptor for inflammatory reactions expressed in leukocytes and other cell types (Barmania and Pepper, 2013). CCR5Δ32 is a 32-base pair deletion in the *CCR5* that, in homozygosis, results in a lack of expression of the functional CCR5 on the cell surface. Heterozygous carriers express lower levels of functional CCR5 compared to wild-type homozygous individuals (Venkatesan et al., 2002). Considering the intense inflammatory response in PE, and based on the high frequency of the deletion allele in healthy pregnant women, Telini et al. (2014) suggested a protective role of the *CCR5*Δ*32* allele against PE development in a Brazilian study.

Adipocytokines are involved in trophoblast invasion and successful placentation. Visfatin is an adipocytokine also known as nicotinamide phosphoribosyltransferase (NAMPT), which has a potential role in the pathophysiology of metabolic disorders such as hypertension and obesity (Dahl et al., 2012). A study by Luizon et al. (2015) evaluated visfatin/NAMPT plasma levels in healthy pregnant women and in patients with gestational hypertension or PE, in the context of the *NAMPT* SNPs −423T < C (rs1319501) and rs3801266A < G in intron 1. No effects were observed concerning rs1319501. Nevertheless, gestational hypertensive patients carrying the rs3801266 “AG” and “GG” genotypes had higher visfatin/NAMPT levels compared to gestational hypertensive patients carrying the “AA” genotype (Luizon et al., 2015). Moreover, Luizon et al. (2017) evaluated whether *NAMPT* SNPs (rs1319501T>C, rs3801266A>G), haplotypes and gene-gene interactions in the NAMPT pathway could affect plasma visfatin/NAMPT levels, and the response to antihypertensive therapy in PE and hypertensive pregnant women. Low circulating visfatin/NAMPT levels were seen in non-responsive PE patients with the rs1319501 TC+CC genotypes. Conversely, high circulating visfatin/NAMPT levels were detected in non-responsive PE patients with the rs3801266 AG+GG genotypes. Haplotype analysis revealed an association of the ‘C, A' haplotype with response to antihypertensive treatment and with low visfatin/NAMPT levels in PE (Luizon et al., 2017). Since lymphotoxin alpha (LTα) is an inflammatory mediator, this molecule was evaluated in the context of PE development, but no association of LTA +252 (rs909253) with PE risk was reported in a Brazilian study (Pissetti et al., 2015).

In order to evaluate the contribution of the inflammasome in PE development, SNPs in the genes coding for nod-like receptors with a pyrin domain 1 (*NLRP1*), *NLRP3*, caspase recruitment domain 8 (*CARD8*), and *IL1B* were studied in a Brazilian population. The *NLRP1* rs12150220A/T SNP was associated with PE. The minor “T” allele was more frequent in PE compared to healthy pregnancy controls, indicating that this allele might be relevant in PE susceptibility. A strong association with *NLRP1* (rs12150550) was also observed in this study, suggesting a role for this molecule in the pathogenesis of PE. Besides, *NLRP1* SNPs produce six main haplotypes, and the rs11651270/C-rs12150220/A-rs2670660/A combination was less frequent amongst PE women compared to controls, suggesting a protective effect against PE (Pontillo et al., 2015).

In the context of gestational hypertension, the relationship between aldosterone levels and SNPs of the aldosterone synthase (*CYP11B2*) gene (−344T/C) and the mineralocorticoid receptor gene (S810L) was investigated in a Mexican population. No differences in genotype distributions or in aldosterone levels were found (Ramírez-Salazar et al., 2011). Similar results were obtained for a Brazilian population (de Vasconcelos et al., 2009).

In summary, several studies covered in this review (Table 1), and other approaches have reinforced that PE is a polygenic disorder and manifests as complex phenotypes, resulting from both maternal and fetal genetic features (Triche et al., 2014). In Latin American populations, conflicting results regarding genetic variants and PE risk were observed, implying that genetic variability does account for this complex phenomenon. Therefore, the search for potential genetic components involved in PE, or its severity, is of paramount importance for a better understanding of the genetic basis of PE pathophysiology (Figure 2). Importantly, we observed a worrying lack of family-based studies evaluating the genetic components of both the fetus/placenta and its biological parents. Thus, such an approach would provide a more actual picture of the genetic risk factors involved in PE and possibly a more accurate disease monitoring and clinical management.

### Vascular and Angiogenic Mediators

Studies also examined gene variants involved in vasculogenesis and angiogenesis, given the importance of establishing an adequate and efficient placental vascular system for a favorable gestational outcome (Herr et al., 2010). Studies from Latin America are summarized in Table 2. Nitric Oxide (NO) has a primary role in the circulatory system. Also, NO is a critical regulatory molecule in ovulation, embryo implantation, pregnancy maintenance, labor, and delivery. Imbalances in NO levels during gestation were suggested as a cause of the development of pregnancy-induced hypertension and PE (Maul et al., 2003). Several studies have evaluated SNPs in both endothelial and inducible nitric oxide synthase genes (*eNOS* and *iNOS*, respectively). In a multicenter study in Colombia, Serrano et al. (2004) evaluated the role of *eNOS* SNPs: Glu298Asp, −786T → C, and VNTR b/a (27 bp-tandem repeat, where “a” and “b” refer to PCR product size, comprising 420 bp for “b” and 393 bp for “a” alleles) as potential risk factors for PE. Young Colombian women homozygous for the Asp298 allele were reported to have an increased risk for PE. The authors suggested that homozygous women for the Asp298 allele are more susceptible to endothelial dysfunction and at increased risk for PE development, since the homozygous state is likely to generate low NO levels. In addition, the presence of the “Asp298-786C-4b” haplotype was associated with an increased PE risk (Serrano et al., 2004). Similarly, Sandrim et al. (2008) compared the same *eNOS* SNPs in women with and without PE from Brazil. Interestingly, the study observed that two *eNOS* haplotypes (“T Glu a” and “C Glu a”) were associated with PE and gestational hypertension. The same SNPs were also evaluated in a Maya-Mestizo Mexican population. The Asp298 allele was associated with PE in a recessive model. In addition, the “−786C-4b-Asp298” haplotype was more frequent in PE than in controls, whereas the “−786T-4b-Asp298” and “−786C-4b-Glu298” haplotypes had lower frequencies or were absent in patients (Díaz-Olguín et al., 2011). In another study, Alpoim et al. (2014) evaluated these same *eNOS* SNPs in early and late severe preeclamptic Brazilian women, and in a group of normotensive/healthy pregnant controls. The frequency of the 894T allele was higher in late severe PE compared to early severe PE. Also, the overall 894T frequency was higher in PE when compared to controls. Regarding the VNTR b/a SNP, higher “aa” genotype and “a” allele frequencies were observed in early severe PE compared to late severe PE and controls. Also, the “T-b-C” haplotype was more frequent in late severe PE compared to early severe PE and controls.

**Table 2:** Summary of studies developed in Latin America evaluating the role of genetic variation in vascular- and angiogenesis-related genes in PE.

**Factors**	**Sample size^†^**	**Key findings**	**Country**	**References**
*eNOS* (−786T>C, intron-4 b/a, Glu298Asp)	322/522	Association with PE risk: 298Asp/Asp genotype and *eNOS* C-b-Asp haplotype.	Colombia	Serrano et al., 2004
*eNOS* (−786T>C, intron-4 b/a, Glu298Asp)	216/110^a^	Association with PE and GH risk: *eNOS* C-a-Glu haplotype. Association with protection for PE and GH: *eNOS* T-a-Glu haplotype	Brazil	Sandrim et al., 2008
e*NOS* (−786T>C, intron-4 b/a, Glu298Asp)	127/263	Association with PE risk: 298Asp/Asp genotype and *eNOS* C-b-Asp.	Mexico	Díaz-Olguín et al., 2011
*eNOS* (−786T>C, intron-4 b/a, Glu298Asp) MMP2 (C-1306T) MMP9 (C-1562T)	77/266	Association with PE risk and severity: −786CC genotype and −786C allele, respectively.	Brazil	Leonardo et al., 2015
*eNOS* (−786T>C, intron-4 b/a, Glu298Asp)	98/103^b^	Association with late-onset PE risk: 298Asp/Asp genotype and 298Asp allele; intron-4 aa genotype and a allele; *eNOS* C-b-Asp.	Brazil	Alpoim et al., 2014
*eNOS* (−786T>C), intron-4 b/a, Glu298Asp	152/152^c^	Association with anti-hypertensive therapy in PE, *eNOS* haplotypes: C-a-Glu responsive and T-a-Asp non-responsive.	Brazil	Sandrim et al., 2010a
*eNOS* (−786T>C, intron-4 b/a, Glu298Asp, rs743506, rs7830)	295/122^d^	Association with protection for PE and GH: eNOS C-b-Glu-G-C haplotype.	Brazil	Muniz et al., 2012
*eNOS* (C-1026A, G2087A)	353/212^e^	Association with PE risk: 2087GA genotype and the 2087A allele.	Brazil	Amaral et al., 2012
*HP* (Hp1-1, Hp2-1, Hp2-2)	92/105	No association with PE risk. Nitric Oxide byproducts in PE associated with Hp2-1 and Hp2-2 genotypes.	Brazil	Sertório et al., 2013
*TAFI* (G505A, C1040T, G-438A)	87/87	No association with PE.	Mexico	Acosta-Tejeda et al., 2011
*MTHFR* (C677T) *FV LEIDEN* (G1691A) PROTHROMBIN (G20210A)	28/41	No association with PE.	Mexico	Rojas et al., 2010
*MTHFR* (C677T)	148/490^f^	No association with PE	Mexico	Pérez-Mutul et al., 2004
*MTHFR* (C677T, A1298C)	150/150	Association with PE risk: 1298CC genotype.	Ecuador	Chedraui et al., 2014
*MTHFR* (C677T)	125/274	Association with protection for PE: 677TT genotype and 677T allele.	Mexico	Canto et al., 2008
*VEGF* (C-2578A, G-1154A, G-634C)	195/108^g^	Association with protection for PE: *VEGF*−2578C/-1154G/-634C haplotype. Low proportion of-2578AA and−634GG genotypes in white PE women.	Brazil	Sandrim et al., 2009
*VEGF* (C936T, C-2578A)	52/28	Association with protection for PE: *VEGF*−2578A allele.	Brazil	Cunha et al., 2011
*VEGF* (C2578A, G634C)	113^h^	No association with PE.	Brazil	Sandrim et al., 2015
*VEGF* (G634C) *IL1A* (rs3783550)	79/210	Association with PE risk: *IL1A* rs3783550 “A” allele.	Brazil	Silva et al., 2015
*VEGF* (A2578C, C1498T, A1154G, C634G,C936T)	31/31^i^	No association with PE.	Ecuador	Chedraui et al., 2013
*eNOS* (T786C, VNTR, G894T) *MTHFR* (C677T) *AGT* (C704T)	230/350	No association with PE.	Mexico	Coral-Vázquez et al., 2013
*MMP9* (C1562T, (CA)n repeats)	300/176^j^	Association with risk for GH: *MMP9* C1562 T allele. No association with PE.	Brazil	Palei et al., 2010
*eNOS* (T786C, VNTR, G894T) *MMP9* (C1562T, (CA)n repeats) *VEGF* (C2578A, G634C)	229/102^k^	Association with protection for PE: combination of MMP9-1562CC with VEGF-634GG genotypes. Association with PE risk: combination of MMP9-1562CC with VEGF-634CC or MMP9-1562CT with VEGF-634CC or-634GG genotypes.	Brazil	Luizon et al., 2012
*MMP2* (C1306T, C735T)	263/130^l^	No association with PE.	Brazil	Palei et al., 2012a
*MMP9* (C1562T, (CA)n repeats)	399/214^m^	Association with GH: combination of the “T” allele for the C1562T and “H” allele of 90(CA)13–25.	Brazil	Palei et al., 2012b
*MTHFR* (C677T) *Factor II* (G20210A) *FV LEIDEN* (G1691A) *PAI1* (4G/5G I/D)	75/145	No association with PE.	Brazil	Dalmáz et al., 2006
*MTHFR* (C677T) *FV LEIDEN* (G1691A)	33/62	No association with PE.	Mexico	Dávalos et al., 2005
*ACVR2A* (rs1424954, rs1014064, rs1424941, rs2161983, rs3768687)	613/693^n^	Association with severe early-onset PE risk: SNPs rs1014064 “G,” rs1424954 “A,” and rs2161983 “A.”	Brazil	Ferreira et al., 2015
*ACE* (287 bp I/D in intron 16)	51/71	No association with PE.	Brazil	Galão et al., 2004
*FV LEIDEN* (G1691A) *Factor II* (G20210A) *MTHFR* (C677T)	30/83	No association with PE.	Brazil	Dusse et al., 2007
*ACE* (287 bp I/D in intron 16)	66/37	Association with risk for PE: *ACE* “DD” genotype.	Mexico	González-Garrido et al., 2017
*EDN1* (G5665T)	61/49^o^	Association with protection for PE: paternal *EDN1* “GG” and “GT” genotypes.	Mexico	Galaviz-Hernandez et al., 2016
*MTHFR* (C677T, A1298C)	50/50^p^	Association with risk for PE: *MTHFR* 677TT genotype.	Ecuador	Chedraui et al, 2015
*ACE* (287 bp I/D in intron 16)	665/1,046	No association with PE.	Colombia	Serrano et al., 2006
*HIF1A* (C1772T, G1790A)	150/105	No association with PE.	Mexico	Nava-Salazar et al., 2011
*VDR* (*FokI, ApaI, BsmI*)	316/213^q^	No association with PE.	Brazil	Rezende et al., 2012
*COMT* (rs6269, rs4633, rs4680, and rs4818), *MTHFR* (C677T)	528/575^r^	Association with PE risk: “ATCA” haplotype of *COMT* (SNPs rs6269, rs4633, rs4818, rs4680, and MTHFR 677T)	Chile	Hill et al., 2011a

† Pooled cases/controls.

a Cases were stratified in PE (n = 113) and gestational hypertension (GH, n = 103).

b Cases were stratified in early severe PE (n = 53) and late severe PE (n = 45).

c Cases were stratified in PE (n = 152) and GH (n = 152).

d Cases were stratified in PE (n = 157) and GH (n = 138).

e Cases were stratified in PE (n = 187) and GH (n = 166).

f Sample size composed by PE cases (n = 148), health pregnant woman (N = 177), and health non-pregnant volunteers (313).

g Cases were stratified in PE (n = 94) and GH (n = 101).

h Sample size was composed by 113 PE white women who were responsive (n = 46) and non-responsive (n = 67) to anti-hypertensive treatment.

i Sample size was composed by 62 cord vessels of singleton gestations with severe PE (n = 31) and controls (n = 31).

j Cases were stratified in PE (n = 154) and GH (n = 146).

k Cases were stratified in PE (n = 122) and GH (n = 107).

l Cases were stratified in PE (n = 133) and GH (n = 130).

m Cases were stratified in PE (n = 214) and GH (n = 185).

n Cases were stratified in PE (n = 443), eclampsia (n = 138), and HELLP syndrome (n = 693).

o °Sample size composed by PE cases (n = 61) and their partners (n = 61), and the control group was health pregnant woman (N = 49) and their partners (n = 49).

p Sample size composed by 100 placental tissues of PE cases (n = 50) and controls (n = 50).

q Cases were stratified in PE (n = 162) and GH (n = 154).

r *Sample size was composed by maternal-fetus dyads from PE cases (n = 528) and controls (n = 575)*.

Although anti-hypertensive treatment has never been demonstrated to reverse PE outcome, its usage could prevent cardiovascular and cerebrovascular adverse consequences, due to severe and rapid elevations of the blood pressure, being a critical tool for clinical PE management. In this sense, it was proposed that anti-hypertensive therapy can enable maintenance of gestation and increase the gestational age of delivery, thus decreasing adverse fetal and maternal outcomes (Podymow and August, 2008). In this context, an elegant study compared the distribution of *eNOS* variants in gestational hypertensive and PE cases who were responsive to anti-hypertensive therapy versus cases who did not respond to treatment. Interestingly, a difference in the overall distribution of *eNOS* haplotypes was observed when PE responsive to treatment groups and PE nonresponsive to treatment groups were compared. The “C Glu a” haplotype was more frequent in the responsive PE group than in the nonresponsive PE group, and the “T Asp a” haplotype was less frequent in the active PE group than in the nonresponsive PE group. This was a pioneer study approaching the genetic background in the context of gestational hypertension treatment (Sandrim et al., 2010b).

The distribution of two *eNOS* Tag SNPs, rs743506 and rs7830, as well as the SNPs T-768C, Intron-4, and G894T, among healthy pregnant controls, gestational hypertensive subjects, and PE subjects was assessed by Muniz et al. (2012). No differences were detected among genotype frequencies in the three groups studied. However, the haplotype H5 “CbGGC” (“C” of rs2070744, “b” of intron 4, “G” of rs1799983, “G” of rs743506, and “C” of rs7830) was more frequent in the control group compared to gestational hypertensive and PE individuals, suggesting a potential protective effect against hypertensive disorders development in pregnancy.

Two *iNOS* SNPs, C-1026A (rs2779249) and G2087A (rs2297518), were evaluated in Brazilian healthy pregnant/control, gestational hypertension, and PE groups. The “GA” genotype and the “A” allele for the G2087A were more commonly found amongst PE subjects. No differences were observed concerning the other variants evaluated (Amaral et al., 2012).

Considering that increased levels of hemoglobin (Hb) and haptoglobin (Hp) complexes contribute to impaired NO bioavailability in PE (Sandrim et al., 2010a), the role of a haptoglobin SNP (duplication of exons 3 and 4 of *HP* gene) was evaluated in PE and non-pregnant women, in the context of NO bioavailability. Higher NO consumption was detected in association with increased cell-free Hb in plasma from PE patients carrying the allele *HP2* (duplicated exons 3 and 4 of the *HP1*), suggesting a functional association between *HP* SNPs and the hemodynamic imbalances observed in PE (Sertório et al., 2013).

Thrombin-activated fibrinolysis inhibitor (*TAFI*) gene has also attracted attention in the context of SNPs and their possible association with vascular disorders in pregnancy. In this scenario, a case-control study investigated the possible association between PE and *TAFI* SNPs (G505A, C1040T, and G-438A), together with TAFIa plasma levels in a Mexican-Mestizo population. No associations with increased PE risk were observed. However, due to higher plasma TAFIa levels and the presence of the G505A mutant genotype, together with wild-type forms of C1040C and G-438G, it was suggested that *TAFI* SNPs in the coding region or in nearby regulatory elements could contribute to variations in TAFIa plasma concentrations (Acosta-Tejeda et al., 2011).

The methylenetetrahydrofolate reductase (MTHFR) enzyme is critical for homocysteine (HCY) metabolism, where it catalyzes the NADPH-linked reduction of 5,10-MTHF to 5-MTHF, and subsequently the methylation of HCY to methionine in a vitamin B12-dependent manner (Barbosa et al., 2008). In a Tunisian study, low MTHFR activity levels were associated with mild to moderate increases in plasma HCY levels in placental vascular complications (Klai et al., 2011). In the same study, the *MTHFR* A1298C variant was associated with recurrent pregnancy loss, intrauterine growth restriction, and placental abruption. In the context of PE, a differential distribution of the *MTHFR* C677T alleles was associated with thrombosis markers and endothelial activation in a study with Mexican women (Rojas et al., 2010). Moreover, a possible association between C677T SNP of *MTHFR* gene and PE was investigated in pregnant women from the Yucatan Peninsula in southeastern Mexico, although no differences between cases and controls were observed (Pérez-Mutul et al., 2004).

In another study evaluating *MTHFR* (C677T) in Maya-Mestizo PE women, it was observed that *MTHFR* “T” allele and the “TT” genotype were more frequent in controls, suggesting a decreased risk of PE in women carrying this variant (Canto et al., 2008). Amongst a Mestizo-Ecuadorian population, the prevalence of C677T and A1298C *MTHFR* SNPs was also investigated in the context of PE, with the “CC” genotype of A1298C occurring in higher prevalence in PE women than controls (Chedraui et al., 2014). Nevertheless, contradictory results regarding PE, the placental genotype, and allele frequencies of the *MTHFR* C677T were observed (Chedraui et al., 2015). Interestingly, for the C677T SNP, the mutant “TT” genotype was threefold more frequent in preeclamptic placentas than controls. In a Chilean population, epistatic interactions between *MTHFR* and catechol-O-methyltransferase (COMT) gene were evaluated in maternal-fetus dyads. The increased PE risk was observed exclusively when the fetus harbored both the *COMT* “ATCA” haplotype (respectively composed by the SNPs rs6269, rs4633, rs4818, rs4680) and the *MTHFR* 677T allele (Hill et al., 2011a).

SNPs in the vascular endothelial growth factor (*VEGF*) gene are also largely studied in PE. Importantly, the low production of VEGF by peripheral blood mononuclear cells is associated with PE (Cardenas-Mondragon et al., 2014). The possible role of SNPs at the promoter region of *VEGF* was addressed by Sandrim et al. (2009). The study reported an association between PE development and the SNPs −2578C/A (rs699947), −1154G/A (rs1570360), and −634G/C (rs2010963). Importantly, inter-ethnic differences account for differential allelic and haplotype distributions, and this is particularly relevant for Latin American populations. In this context, the authors observed that *VEGF* −2578A and −1154A alleles were more frequent in European-descendants subjects compared to Afro-descendants, while no inter-ethnic differences were observed regarding the G-634C SNP. Ethnic origin is also correlated with differences in *VEGF* haplotypic frequencies (Muniz et al., 2009). Cunha et al. (2011) evaluated *VEGF* variants +936C/T and −2578C/A in PE cases and controls. The *VEGF* −2578A allele showed a higher frequency in the control group, suggesting a possible protective effect against PE, while no association of *VEGF* +936C/T was observed in PE or controls (Cunha et al., 2011).

In the context of antihypertensive therapy, *VEGF* SNPs (C-2578A and G-634C) were evaluated in European-derived Brazilian women with PE classified according to response to antihypertensive therapy. No associations were observed, suggesting that these *VEGF* SNPs does not influence the antihypertensive therapy responsiveness in PE (Sandrim et al., 2015). The *VEGF* G-634C and *IL1A* (rs3783550) SNPs were evaluated in Brazilian women with PE and in controls. An association between *IL1A* (rs3783550) SNP and PE development was observed in this population sample. However, no differences were observed regarding the *VEGF* G-634C variant (Silva et al., 2015).

An elegant study investigated *VEGF* SNPs (−2578 A/C, −1498 C/T, −1154 A/G, −634 C/G, and +936 C/T) in samples from cord vessels of singleton gestations with severe PE. Additionally, they investigated NO plasmatic levels, asymmetric dimethyllarginine and VEGF levels in fetal circulation. The SNPs showed similar distributions in cases and controls. Significantly higher NO plasma levels in umbilical vessels were seen in PE. Arterial VEGF levels were significantly lower in PE cases, and a positive correlation was found between NO and asymmetric dimethylarginine levels amongst PE cases (Chedraui et al., 2013).

The influence of SNPs in *eNOS, MTHFR, GSTP1*, and angiotensinogen (*AGT*) genes on PE was evaluated by Coral-Vázquez et al. (2013). The *eNOS* variants covered in the study were: −786T → C (rs2070744), VNTR (27 bp) in intron 4, and G-894T → Glu298Asp (rs1799983). The C-704T → Met235Thr (rs699) was the variant studied in *MTHFR*, the C-704T → Met235Thr (rs699) in *AGT*, and the A-313G → Ile105Val (rs1695) in *GSTP1*. No differences in the distribution of the genotypes or haplotypes between controls and PE cases were observed.

Matrix metalloproteinases (MMPs) are enzymes responsible for the degradation of various extracellular matrix molecules. In pregnancy, a disturbance in MMP activity could indicate abnormal trophoblast invasion. Moreover, detection of MMP up-regulation could reflect an interaction between oxidative stress and inflammatory mediators, which could result in the delivery of cell debris in maternal circulation and accumulation in various maternal organs (Chen and Khalil, 2017). The involvement of MMPs in vascular disorders of pregnancy may worsen the response to antihypertensive therapy (Palei et al., 2012a). In this context, a study investigated two matrix metalloproteinase 9 (*MMP9*) SNPs, the g.−1562C>T (rs3918242) and microsatellite g.−90(CA)13-25 (rs3222264). They report an association of the *MMP9* SNP with gestational hypertension, but not with PE (Palei et al., 2010).

The association of PE and SNPs of nitric oxide synthase 3, *NOS3* (G894T, T-786C, and a VNTR with intron 4), *MMP2* (C-1306T), and *MMP9* (C-1562T) genes was investigated through a prospective case-control study in a southeastern Brazilian population. No association with PE development was found regarding *MMP2* and *MMP9* variants. Considering the *NOS3* gene, the SNP T-786C showed association with PE development (Leonardo et al., 2015). Luizon et al. (2012) evaluated whether epistatic interactions among seven clinically relevant SNPs of *eNOS* (T-786C, rs2070744, a VNTR in intron 4, and Glu298Asp, rs1799983), *MMP-9* [C-1562T, rs3918242 and −90(CA)13-25, rs2234681] and *VEGF* (C-2578A, rs699947, and G-634C, rs2010963) could be associated with PE or gestational hypertension. Significant interactions between the *MMP9* and *VEGF* genes were seen in PE samples (Luizon et al., 2012). The *MMP2* SNPs: g-1306 C>T (rs243865) and g-735 C>T (rs2285053) were analyzed in the context of both PE and gestational hypertension together with circulating MMP-2 and tissue inhibitor of metalloproteinase (TIMP)-2 levels. High MMP-2/TIMP-2 ratios were observed in gestational hypertensive patients, but no differences in the genotype and allelic frequencies were found (Palei et al., 2012a). The same above-mentioned approach was used regarding *MMP9* SNPs: g.−90(CA)13-25 (rs3222264) and g.−1562C>T (rs3918242) and circulating levels of MMP-9 and TIMP-1. Higher plasma concentrations of MMP-9 and TIMP-1 were detected in gestational hypertensive patients compared to controls. TIMP-1 levels were higher in PE cases, but MMP-9 and MMP-9/TIMP-1 ratios were similar between PE and gestational hypertensive subjects. Haplotype analyses suggested that the presence of the H4 haplotype increases susceptibility to gestational hypertension (Palei et al., 2012b).

Dalmáz et al. (2006) assessed the prevalence of four thrombophilic genes in women with mild or severe PE in Southern Brazil. Variants studied include the *MTHFR* C677T, prothrombin gene (*F II*) G20210A, *Factor V* (*FV Leiden*) G1691A, and insertion/deletion (4G/5G) in the plasminogen activator inhibitor type 1 (*PAI1*) gene promoter region. No association between PE and the SNPs was observed (Dalmáz et al., 2006). *MTHFR* C677T and *Factor V Leiden* SNPs were also investigated as potential genetic risk factors for eclampsia and PE in a group of women from western Mexico, without statistically significant results (Dávalos et al., 2005). Furthermore, *Factor V Leiden* (G1691A), *Factor II* (G20210A), and *MTHFR* (C677T) variants were investigated in the context of inherited thrombophilia in Brazilian PE women and controls. Again, no differences were observed (Dusse et al., 2007).

The gene *ACVR2A* encodes the activin A type II receptor (ActRIIA), an essential factor for pregnancy establishment during decidualization, trophoblast invasion, and placentation. Concerning the regulation of trophoblast invasion, abnormal decidual *ACVR2A* expression may affect placentation and lead to PE development (Yong et al., 2018a). In this context, five *ACVR2A* SNPs (rs1424954, rs1014064, rs1424941, rs2161983, and rs3768687) were investigated in a northwestern Brazilian population approaching PE cases and controls. These five SNPs showed no association with PE. Nevertheless, haplotype analysis revealed a strong association among SNPs rs1014064, rs1424954, and rs2161983 and severe early-onset PE (Ferreira et al., 2015).

Considering that the cardiovascular system of a pregnant woman adapts to allow and support increased blood flow toward the placenta, angiotensin-converting enzyme gene (*ACE*) SNPs were investigated in vascular disorders of pregnancy. A common 287-bp insertion/deletion SNP within *ACE* (*ACE*-I/D) was investigated as a possible risk factor for PE development in a south Brazilian population. The allele frequencies of this *ACE* variant were not associated with PE development (Galão et al., 2004). Subsequently, a case-control study and meta-analysis were also unable to show the association between the *ACE*-I/D variant and PE (Serrano et al., 2006). In a Mexican population, González-Garrido et al. (2017) evaluated the *ACE*-I/D SNP in relation to ACE activity and oxidative damage in PE. Higher ACE activity was found in PE cases compared to controls. Also, higher “DD” genotype and “D” allelic frequencies were found in PE compared to the control group. In summary, the results suggested that *ACE*-I/D SNP, high ACE activity, body mass index and oxidative damage are important factors in the pathogenesis of PE (González-Garrido et al., 2017).

The Endothelin 1 protein is a potent vasoconstrictor molecule, and its encoding gene, *END1*, is also a candidate gene for PE. A case-control study investigated women affected with PE and their partners in comparison to healthy pregnant women and their partners regarding the *EDN1* rs5370 SNP. A negative association between the rs5370 SNP and PE in the male sub-group was observed, while no association was observed between cases and controls in the female sub-group (Galaviz-Hernandez et al., 2016). This study reminds us of the importance of including the paternal genetic background and the effect of the male genetic contribution in pregnancy outcomes.

Hypoxia-inducible factor (HIF) is a highly conserved transcription factor that coordinates an adaptive response in physiological and pathophysiological situations. Several cell types up-regulate *HIF* in response to low oxygen levels. In human pregnancy, HIF signaling in the gravid uterus is critical for fetal and placental development (Macklin et al., 2017). The Influence of *HIF1A* C1772T and G1790A SNPs was evaluated in PE patients and controls in a Mexican population, although no association of these variants with PE risk was observed (Nava-Salazar et al., 2011).

Finally, vitamin D is an essential molecule during pregnancy. The levels of its active form increase throughout healthy gestation and are critical for an adequate calcium supply for fetal growth (Urrutia and Thorp, 2012). Given its importance and relevance in gestational outcome, Vitamin D Receptor (*VDR*) SNPs have been studied in PE and gestational hypertension. Rezende et al. (2012) evaluated three *VDR* SNPs (*FokI, ApaI*, and *BsmI*) in a Brazilian population, and also investigated the potential association of hypertensive pregnancy disorders with *VDR* haplotypes. No differences in genotype, allele, or haplotype frequencies were observed between PE or pregnant hypertensive women and controls, these findings suggested that the investigated SNPs do not influence pregnancy outcome (Rezende et al., 2012).

Studies in vascular and angiogenic gene polymorphisms have shown conflicting results in Latin American populations (Table 2). Besides genetic variants alone, PE-associated haplotypes and the interaction among SNPs of distinct genes further support the importance of exploratory studies in this rapidly developing field. The conflicting results evidenced in this review are partially explained by the differences in the genetic background of distinct Latin American populations, which result from high admixture (Salzano and Sans, 2014). Besides, the different genotypic and allelic frequencies of the studied SNPs corroborate the PE classification as a complex disease. For a better understanding of the whole scenario involving this disease, robust studies and several exploratory studies still need to be put into practice. Also, the publication of negative results is important, mainly for the correct performance of meta-analyses encompassing preexisting data that would better reflect the actual frequency of genetic variants in Latin American populations.

## Genetic Variation in Histocompatibility-Related Genes in PE

The major histocompatibility complex (MHC) is fundamental to the immunological system allowing the development of immune responses against foreign antigens or immunogenic epitopes through recognition of self- and non-self. Traditionally, the MHC complex is defined by two well-known genetic *loci*: MHC class-I and MHC class-II, although MHC class-III and -IV also exist and are relevant to complex diseases (Gruen and Weissman, 2001; Yau et al., 2016). MHC class-I members split in “classical” [human leukocyte antigen (*HLA*)-*A*, -*B*, and -*C*] and “non-classical” (*HLA-E*, -*F*, -*G* and -*H*: or MHC-Ib) molecules. Classical genes are ubiquitously expressed on virtually all nucleated cells (with a few exceptions), are highly polymorphic, and their primary function is as peptide presenting molecules. On the contrary, expression of non-classical molecules are restricted to some cellular types (for example, EVT), have a limited degree of polymorphism, and do not present peptides as a major function but rather act as signaling molecules to immune cells. Classical MHC class-II (*HLA-DR*, -*DQ*, and -*DP*) expression is restricted to antigen presenting cells, such as B cells, macrophages, and DCs. MHC class-III and IV are otherwise very distinct molecules comprising members of the complement system and induced-stress/inflammatory proteins, respectively (Gruen and Weissman, 2001).

The role of the MHC-Ib molecules in pregnancy has been a focus since the discovery of HLA-G expression in human trophoblast cells (Kovats et al., 1990). In the maternal-placental interface, an exciting aspect is the expression of HLA-G, -E, -F, and -C antigens on EVT cells (Hackmon et al., 2017).

Among the non-classical MHC-I molecules, HLA-G is a most enigmatic member. It interacts with several maternal immune cells, including those in the decidua (i.e., dNK, decidual macrophages, dCD4+, dCD8+), and has the potential to inhibit or activate their immunologic functions. Recently, it was reported that soluble HLA-G (sHLA-G) affinity for its cognate receptors [i.e., dimers binding to LILRB1 (leukocyte Ig-like receptor 1) with increased affinity] is likely impacted by sHLA-G heterodimerization in inflamed patients, which is likely to occur in PE and explains the variable findings reported so far (Veit et al., 2015). Also, LILRB1 receptors bind to β2-microglobulin(m)-associated HLA-G, whereas the LILRB2 receptors bind to non-β2m-associated HLA-G molecules. Alternative splicing of the gene results in seven isoforms: four membrane-bound HLA-G isoforms (HLA-G1 to -G4) and three soluble isoforms (HLA-G5 to G7). HLA-G1 undergoes proteolytic cleavage by metalloproteinase-2 (MMP-2) giving rise to sHLA-G1 (Rizzo et al., 2013).

In Latin America, several groups have evaluated the role of candidate genes belonging to the MHC loci and PE susceptibility (Table 3). *HLA-G* is the most studied *MHC* gene due to its immunotolerogenic properties, and several aspects of *HLA-G* have been explored. An SNP located in the 3′ untranslated region (UTR) of the gene, namely 14-bp insertion(ins)/deletion(del) (rs66554220), is well-known due to its influence on mRNA stability which affects the expression patterns of the gene (Rousseau et al., 2003; Porto et al., 2015). We have recently reported that specific haplotypes and variants in the 3′UTR increase the risk for recurrent pregnancy loss in Brazilian women (Michita et al., 2016). Also, we suggested that a maternal 14 bp del/del homozygous status might predispose primiparous women to PE (Vianna et al., 2007). An increased risk for PE was also observed in neonates who preferentially inherit the maternal HLA-G^*^0104 allele (Carreiras et al., 2002), which has been associated with the 14 bp del allele present in the UTR-3 haplotype (Castelli et al., 2014). In another study, a concomitantly low frequency of CD8+CD28- T cells (CD8+T memory cells), low monocyte (CD14+HLA-G+), and low T cell (CD3+HLA-G+) counts in PE women were associated with a pro-inflammatory status, which was confirmed by pro-inflammatory cytokine measurements (Vianna et al., 2016); however, no differences in 14 bp ins/del and +3142C/G (rs1063320) SNP frequencies between PE and non-PE women were observed. Similarly, in a Mexican PE cohort, although HLA-G expression was not evaluated, a reduced frequency of CD3+ T cells was observed in third trimester decidual tissue, and most importantly, dNK cells (CD3-CD56+CD16-CD9+) persisted throughout pregnancy and shared the same phenotype as the ones detected in early pregnancy (Sánchez-Rodríguez et al., 2011). This implies that long-term persistence of dNK cells could play important physiological roles in labor by the secretion of inflammatory mediators and fighting against infectious agents. Still considering HLA-G, Ferreira et al. (2017) reported that the 14 bp variant had no influence on PE predisposition, although the specific contribution of this SNP for PE in primiparous women was not evaluated. Hitherto, the role of the 14 bp variant in PE has been a matter of debate (Vianna et al., 2007; Pabalan et al., 2015; Ferreira et al., 2017). However, in a recent meta-analysis, the ethnicity (European-derived) and the 14 bp ins/ins genotype status in neonates were pointed as likely involved in PE risk in primiparous women (Pabalan et al., 2015).

**Table 3:** Summary of studies in Latin America evaluating the role of genetic variants in histocompatibility-related genes in PE.

**Factors**	**Sample size^†^**	**Key findings**	**Country**	**References**
*HLA*-*A*, -*G*, -*DRB1*, -*DQA1*, -*DQB1* alleles	27/29^a^	Association with PE risk: HLA-G*0104 allele, DRB1*07 DQA1*0201 DQB1*0201 haplotype and DRB1*07 and/or DRB1*06 alleles in presence of HCMV detection.	Venezuela	Carreiras et al., 2002
*HLA-G* (14 bp ins/del)	157/162	No association with PE.	Brazil	Vianna et al., 2007
*KIR* inhibitory(*2DL1, 2DL2, 2DL3, 2DL4, 2DL5, 3DL1, 3DL2, 3DL3*); activating (*2DS1, 2DS2, 2DS3, 2DS4, 2DS5, 3DS1*); pseudogenes (*2PQ1, 3DP1*)	90/86	No association with PE.	Mexico	Sánchez-Rodríguez et al., 2011
*HLA-G* (14 bp ins/del, +3142C>G).	26/32^b^	No association with PE.	Brazil	Vianna et al., 2016
*HLA-G* (14 bp ins/del)	409/332^c^	No association with PE.	Brazil	Ferreira et al., 2017
*ERAP2* (rs2549782, rs17408150)	528/575^d^	No association with PE.	Chile	Hill et al., 2011b

† Pooled cases/controls.

a Samples were mother-neonate dyads.

b Controls were grouped in non-PE (n = 25) and healthy group (n = 7).

c Cases were grouped in PE (n = 246), eclampsia (n = 57), and HELLP (n = 106). PE, preeclampsia; HLA, human leukocyte antigen; HCMV, human cytomegalovirus; ins, insertion; del, deletion; KIR, killer cell immunoglobulin-like receptor; ERAP2, endoplasmic reticulum aminopeptidase-2

d *Only Chilean mother-neonate dyads*.

PE development probably involves the interaction of maternal and fetal features. Also, a contribution of paternal origin has been suggested (Dahl et al., 2014; Saftlas et al., 2014). Functional variants within the endoplasmic reticulum aminopeptidase gene (*ERAP2*) have been associated with PE in non-Latin American populations (Johnson et al., 2009). In a study by Hill et al. (2011a), the *ERAP* variants rs2549782 and rs17408150 were evaluated in Chilean dyads (mother-neonate) and African American subjects (78% were dyads) with PE. In this study, no influence of *ERAP* SNPs in PE predisposition was reported. The lack of association with PE risk could be partially explained by differences in population structure and linkage disequilibrium patterns (Hill et al., 2011b). A study evaluating Venezuelan dyads reported an increased risk for PE in both mothers and neonates carrying the HLA-DRB1^*^07 DQA1^*^0201 DQB1^*^0201 haplotype. In addition, mothers carrying the HLA-DRB1^*^06/07 allele were more likely to be infected by the human cytomegalovirus (HCMV) (Carreiras et al., 2002). Since the recent Zika virus epidemics in Brazil (Schuler-Faccini et al., 2016), the relevance of viral infections during pregnancy is once more in the spotlight. Of note, recent evidence suggests that some viral infections modify the threshold of placental cell immunologic response to bacterial lipopolysaccharides (LPS) resulting in an exacerbated inflammatory response, and thus contributing to the development of pregnancy disorders including PE (Cross et al., 2017; Nourollahpour Shiadeh et al., 2017).

Other polymorphic loci immunologically relevant in PE comprise the *KIR* (Killer-cell immunoglobulin-like receptors) family. This gene family encompasses both activating (S) and inhibitory (L) receptors and can be functionally characterized in two additional groups: A (inhibitory) or B (activating) group. There is evidence of maternal *KIR* contribution in PE development. Indeed, it was suggested that the predominance of inhibitory receptors in PE women conferred an increased risk for PE in Mexican women (Sánchez-Rodríguez et al., 2011). Interestingly, a higher frequency of CMV-positivity was observed in third trimester Mexican women carrying the inhibitory *KIR* bB03|tA01 haplotype (*KIR* A) (Alvarado-Hernández et al., 2016), reinforcing the theory that imbalances between activating and inhibitory receptors expressed on cytotoxic cells influence viral infection predisposition and are possibly a risk-modifying factor for pregnancy disorder development.

## Gene Variants Involved in Metabolic Processes

Changes in maternal metabolism occur during gestation, allowing adaptation to the energetic and nutritional needs of the developing fetus and ensuring its healthy development. Some changes involve the metabolism of carbohydrates and lipids. Such metabolic changes occur in a spatial and temporal manner as pregnancy develops. Early in gestation, glucose and insulin levels are comparable to those of non-pregnant women, with a slight increase in insulin sensitivity (Butte, 2000). A decrease in insulin sensitivity occurs naturally, becoming evident in the second trimester, however, a noticeable loss of insulin sensitivity can lead to systemic resistance, hyperglycemia, and gestational diabetes mellitus (DM). The effects of hyperglycemia in pregnancy are associated with several adverse clinical outcomes for both mother and newborn, the latter associated with overweight and cardiometabolic risk later in life (Thaware et al., 2015; Zhu et al., 2016; Tam et al., 2017). It was suggested that gestational hyperglycemia or pre-pregnancy DM are risk factors for gestational disorders, including PE (Wendland et al., 2008). Interestingly, the expression of cytokines (i.e., IL-10 and TNF-alpha) relevant to the pathophysiology of PE (Daher et al., 2006; Pinheiro et al., 2015) is associated with maternal glycemia (Moreli et al., 2012), implying that maternal glycemia not only affects the metabolic status but also the immunological profile of pregnant women.

Some studies have evaluated the role of critical mediators in metabolic processes and their influence on PE development (Table 4). Adiponectin (ADIPOQ) is an adipokine, a term referring to adipose tissue-derived signaling molecules with broad biological functions (Ruan and Dong, 2016). ADIPOQ enhances cellular insulin sensitivity and thus is involved in adipose tissue expansion. Besides metabolic signaling, ADIPOQ has anti-inflammatory, anti-atherogenic and anti-proliferative functions, but paradoxically it is associated with coronary diseases (Sattar and Nelson, 2008). In addition, it enhances human EVT cell invasion *in vitro* by means of MMP-9 and -2 expression and TIMP-2 repression (Benaitreau et al., 2010). Expression of both MMPs in EVT cells may increase membrane cleavage of the immunomodulatory molecules MIC-A and HLA-G (Sun et al., 2011; Rizzo et al., 2013). SNPs in *ADIPOQ* influence basal expression of the gene and predispose occurrences of metabolic disorders in French and Japanese populations (Hara et al., 2002; Fumeron et al., 2004). In a cohort of Brazilian PE women *ADIPOQ* variants −11391G/A (rs17300539), −11377C/G (rs266729), 45T/G (rs2241766), and 276G/T (rs1501299) were evaluated. The rs266729 GG genotype presented a higher frequency in PE (Machado et al., 2014). The −11377G allele is suggested to decrease the affinity of nuclear proteins in the *ADIPOQ* promoter and putatively the transcriptional activity (Bouatia-Naji et al., 2006; Wang et al., 2009; Zhang et al., 2009). Therefore, preeclamptic -11377GG genotype carriers are likely to express low levels of adiponectin, resulting in impaired control of glycemia. Also, −11377G allele carriers have been associated with chronic hypertension (Ong et al., 2010), recurrent pregnancy loss (Dendana et al., 2018), and gestational diabetes (Pawlik et al., 2017) in non-Latin American populations.

**Table 4:** Summary of studies in Latin America evaluating the role of genetic variants within genes involved in metabolic changes during pregnancy.

**Factors**	**Sample size^†^**	**Key findings**	**Country**	**References**
*ADIPOQ* (-11391G>A,−11377C>G, 45T>G, 276G>T)	240/161^a^	Association with PE risk: −11377GG genotype.	Brazil	Machado et al., 2014
*INS* (*Pst*I, *Mae*III) *INSR* (*Nsi*I) *IRS1* (Ala513Pro, Gly972Arg)	43/46	No association with PE.	Mexico	Machorro-Lazo et al., 2009
*LEP* (G2548A) LEPR (Gln223Arg, Lys109Arg)	146^b^	Association with GH clinical findings: LEP 2548AA genotype with BMI and 2548G allele with systemic BP; LEP 109 Lys/Lys genotype with BMI and Insulin resistance.	Brazil	Farias et al., 2017
*LIPC* (-514C>T)	157/180	Association with PE risk: *LIPC* −514TT genotype in overweight pregnant women.	Peru	Enquobahrie et al., 2005

† Pooled cases/controls.

a Cases were grouped in PE (n = 127) and gestational hypertension (n = 113).

a *Prospective cohort of pregnant women. PE, preeclampsia; ADIPOQ, adiponectin; INS, insulin; INSR, insulin receptor; IRS1, insulin receptor substrate-1; LEP, leptin; LEPR, leptin receptor; GH, gestational hypertension; BP, blood pressure; BMI, body mass index; LIPC, hepatic lipase*.

Lipid metabolism and plasmatic concentration are regulated by an enzyme encoded in lipase hepatic gene *LIPC*. *LIPC* −514C/T (rs1800588) is a promoter SNP which influences hepatic lipase levels. In fact, the −514TT genotype is associated with the lowest enzyme activity, although the variant effect is variable among non-Latin American populations (Tahvanainen et al., 1998; Ordovas et al., 2002; Isaacs et al., 2004). This variant was evaluated in a cohort of PE Peruvian women (Enquobahrie et al., 2005). Although no direct association with PE risk was observed, overweight status during pregnancy was a modifying risk factor for PE in *LIPC* −514TT genotype.

Changes in insulin responsiveness are essential in pregnancy and affect both mother and fetus. As pregnancy develops, maternal insulin resistance increases, which in turn facilitates glucose transport across the placenta and stimulates fetal insulin production, favoring normal fetal growth and development (Farrar, 2016). Hyperinsulinemia is harmful and resembles the endothelial dysfunction observed in PE pathophysiology (Muniyappa and Sowers, 2014). An interesting Mexican study evaluating the role of genetic variants of genes involved in insulin responsiveness in PE development focused on: insulin [(*INS*); *Pst*I (rs3842752) and *Mae*III (rs689)], insulin receptor [(*INSR*); *Nsi*I (rs2059806)], and insulin receptor substrate [(*IRS1*); Ala513Pro (rs1801276) and Gly972Arg (rs1801278)] (Machorro-Lazo et al., 2009). Although no statistical difference in SNPs frequencies was observed, a previous study evaluating different ethnic groups in Mexico observed differences in the *Mae*II, *Pst*I, and *Nsi*I genotype distribution when stratified by fasting insulin and serum triglyceride levels (Flores-Martínez et al., 2004; Sánchez-Corona et al., 2004). Also, the *IRS1* 972Arg allele was associated with gestational diabetes in a meta-analysis (Zhang et al., 2013) and the *INSR Nsi*I SNP (rs2059806AA genotype) was associated with PE in an Australian cohort and also in PE newborns small for the gestational age in a Sinhalese cohort (Andraweera et al., 2017). The lack of association with PE is possibly due to the stringent inclusion/exclusion criteria of the study since pregnant women with undiagnosed insulin resistance before pregnancy were excluded.

As insulin signaling involves an intricate network of molecules, it is unlikely that a single gene or SNP results in an insulin-resistant phenotype. Nevertheless, SNPs in leptin (*LEP*) and leptin receptor (*LEPR*) genes seem to have the potential to influence blood pressure during pregnancy as an indirect effect on insulin sensitivity and BMI, and therefore are relevant in PE pathophysiology (Fan and Say, 2014; Taylor et al., 2015). In a Brazilian study, *LEP* G2548A (rs7799039), *LEPR* Q223R (rs1137101), and K(Lys)109R(Arg) (rs1137100) variants were evaluated regarding their influence on maternal blood pressure during pregnancy and the postpartum period (Farias et al., 2017). Although no association with leptin levels and SNPs were observed, homozygous individuals for 2548AA genotype had lower BMI in early pregnancy, and the effect of BMI on blood pressure levels was higher in 2548AA homozygous carriers compared to G allele carriers (GA+GG). On the contrary, 2548GG+GA showed a positive increase in systemic blood pressure in early pregnancy. In a more recent study, the 2548A allele was associated with an increased risk for gestational weight gain (Martins et al., 2017). The influence of G2548A SNP in leptin levels during pregnancy is still not evident (Sugathadasa et al., 2010; Yang et al., 2016; Farias et al., 2017). Nevertheless, in non-pregnant Brazilian women, associations with obesity risk and increased leptin levels for 2548GG genotype and 2548G allele were reported (Hinuy et al., 2008). In PE, plasma levels of leptin are higher than in normotensive pregnant women (Sugathadasa et al., 2010). Also, women with impaired fasting glucose have higher levels of both insulin and leptin compared to euglycemic pregnant women (Yang et al., 2016). These observations are relevant since leptin-induced obesity is associated with hyperglycemia, hypertension, and endothelial damage.

## Variants in Detoxification, DNA-repair, and Apoptosis-Related Genes

Vascular dysfunction is one hallmark of PE that is intensified by positive feedback involving altered maternal immune tolerance and placental hypoxia. In addition, endothelial damage observed in PE is the *prima facie* of impaired clearance of oxidative stress byproduct by endogenous detoxifying agents. Oxidative stress causes membrane lipid peroxidation, DNA damage and is possibly implicated in the pathogenesis of essential hypertension (González, 2014). Functional SNPs in candidate genes of the detoxification system, DNA repair, and apoptosis genes have been suggested to play roles in PE development (Table 5). Glutathione-S-transferase (GST) is an endogenous detoxifying enzyme superfamily that protects against oxidative stress and exogenous toxins or xenobiotics. The functional variant *GSTP1* 313A/G (rs1695) lies within the active site of the GSTP1 enzyme, and the 313G allele (valine) is associated with low catalytic activity (Ali-Osman et al., 1997). Studies evaluating this variant in different continental cohorts of PE have reported conflicting results (Zusterzeel et al., 2000; Gerhardt et al., 2004; Canto et al., 2008; Coral-Vázquez et al., 2013; Gao et al., 2016). On the one hand, it was observed that *GSTP1* 313G allele and 313GG/AG genotypes are protective factors for PE development in Maya-Mestizo women (Canto et al., 2008), a finding inconsistent with a Dutch study (Zusterzeel et al., 2000). This same variant had no influence on severe PE development in Mexican-Mestizo women (Coral-Vázquez et al., 2013), highlighting differences in results according to ethnic origin. Studies evaluating the role of *GSTP1* 313A/G in PE risk reported conflicting results, probably due to the high inter-variation and intra-variation (i.e., admixture) of the *GSTP1* 313G allele frequency (Zerbino et al., 2018). Another interesting *GST* variant is the complete deletion of *GSTM1* and *GSTT1* (Anvar et al., 2011). It is reported that Mexican-Mestizo women homozygous for *GSTT1* null genotype have a higher risk for PE, and those double homozygous for both *GSTM1* and *GSTT1* null genotypes have a 5-fold increased risk for PE (Sandoval-Carrillo et al., 2014a). These findings contribute to the conflicting body of evidence as pointed out by a meta-analysis (Anvar et al., 2011; Ge et al., 2015). Although the frequency of single deletions varies (Palma-Cano et al., 2017), we hypothesized that populations showing a high frequency of both *GSTs* deletions could have a high frequency of individuals carrying both deletion alleles, implying an increased risk to oxidative stress-related disorders such as PE or vasculopathies.

**Table 5:** Summary of the studies in Latin America evaluating the role of genetic variants in genes involved in detoxification, DNA repair and apoptosis in PE.

**Factors**	**Sample size^†^**	**Key findings**	**Country**	**References**
*GSTP1* (313A>G)	125/274	Association with protection for PE: 313GG and AG genotypes.	Mexico	Canto et al., 2008
*GSTP1* (313A>G)	230/352	No association with PE.	Mexico	Coral-Vázquez et al., 2013
*GSTM1, GSTT1*	112/233	Association with PE risk: *GSTT1* deletion, and combined *GSTM1/GSTT1* deletion (highest risk).	Mexico	Sandoval-Carrillo et al., 2014a
*APEX1* (Asp148Glu) *XPD* (Lys751Gln) *XRCC* (Arg399Gln) *XRCC3* (Thr241Met)	202/350	Association with PE risk and disorder severity: *APEX1* 148Glu allele.	Mexico	Sandoval-Carrillo et al., 2014b
*TP53* (Arg72Pro) *MDM2* (309T>G)	119/99	No association with PE.	Brazil	Busatto et al., 2015
*CASP-8* (*rs13416436, rs2037815*)	55/162	No association with PE.	Brazil	Orlando et al., 2018

† *Pooled cases/controls. PE, preeclampsia; GSTP, glutathione s-transferase Pi-1; GTSM1, glutathione s-transferase Mu-1; GSTT1, glutathione s-transferase Theta-1; APEX1, Apex nuclease 1; XPD, Xeroderma pigmentosum complementation group D; XRCC, x-ray repair cross-complementing protein; XRCC3, x-ray repair cross-complementing protein 3; TP53, tumor protein p53; MDM2, mouse double minute-2 homolog; CASP8, caspase-8*.

Most DNA damage caused by endogenous ROS generated from oxidative stress is corrected by the DNA repairing machinery through diverse pathways (see Chatterjee and Walker, 2017). It is not clear whether DNA damage is an effect or cause of PE pathophysiology, although impaired DNA repair is observed in placental tissue from PE women (Tadesse et al., 2014). Also, accumulation of DNA errors results in cell death, and DNA repair efficiency is impacted by genetic variation in DNA repair genes. Hitherto, few studies have investigated such variants in PE development (Vural et al., 2009; Saadat et al., 2012; Sandoval-Carrillo et al., 2014b). In a study enrolling Mexican women with PE, SNPs in DNA repair genes from nucleotide and base excision pathways, homologous recombination and single-strand break repair mechanisms were evaluated. Among the variants evaluated, a possible role for the functional variant T1349G (Asp148Glu; rs1130409) in the apurinic/apyrimidinic (AP) endonuclease (*APEX1*) gene in PE development was observed. Although no difference in overall genotype distribution between PE and normotensive pregnant women was observed, consistent with a previous study (Vural et al., 2009), the 1349G (148Glu) allele frequency was higher in PE subjects compared to normotensive women. Also, the G allele frequency was higher in severe PE compared to mild-PE (Sandoval-Carrillo et al., 2014b). Although a functional study reported no difference in endonuclease activity between APEX1-148Glu and APEX1-148Asp molecules (Hadi et al., 2000), the role of this variant in PE is supported by impaired enzyme functionality (impaired DNA-binding and endonuclease activity) associated with the 1349G (148Glu) allele (Almutairi et al., 2015), and also by the fact that APEX-1 is essential for the base excision repair pathway, apoptosis, response to oxidative stress, and cell cycle control.

Essential for genomic stability and cell cycle control, the tumor suppressor protein p53 is also implicated in human reproduction (Kang and Rosenwaks, 2018). Our research group has investigated the role of the *TP53* Arg72Pro (rs1042522) and *MDM2* 309T/G (rs2279744) variants in PE development (Busatto et al., 2015). Despite a lack of association with PE risk in our study, it is reported that *MDM2* 309GG genotype confers an increased risk for PE in an Iranian population (Salimi et al., 2017). Interestingly, MDM2 309G allele frequency in normotensive and PE women was similar in both studies, although genotypic frequencies differed. These findings highlight that interaction among SNPs from the regulatory *TP53* network are likely to account for observed differences and should be addressed in further studies (Jacovas et al., 2015). Genetic variants in apoptosis-related genes, such as *CASPASE-8* (rs13416436T/A and rs2037815G/A) were evaluated in PE in a small cohort of Brazilian women, although no association with disorder risk was observed (Orlando et al., 2018).

## Future Directions: Challenges and Perspectives

Over the past decade, our understanding of the molecular basis of many disorders has increased in an unprecedented manner. Despite improvements in understanding the contribution of paternal, maternal, and placental factors in PE pathophysiology, the identification of reliable predictive biomarkers for PE remains elusive. We do not wish to distract from the importance and biological implications of the many other advances in PE understanding, however, based on our knowledge we suggest future directions/studies and challenges in PE research by highlighting and discussing some emerging trends from distinct but related biological fields (Figure 2).

### MHC Class-I Related Sequence A

It is well known that some biological aspects inherent to host immunologic tolerance to solid organ allograft transplantation (tx) could overlap to some extent with those directly related to human pregnancy (sometimes considered as a naturally occurring grafting event). Relevant in human pregnancy, the MHC Class-Ib molecules are becoming a target of studies in human transplantation, since the rejection of allografts fully matched for HLA antigens still occur. In this context, the non-classical MHC class-I related sequence A (MIC-A or MICA), a stress-induced protein has attracted attention due to its immunomodulatory properties (Baranwal and Mehra, 2017; Risti and Bicalho, 2017). MICA has restricted tissue expression in normal physiological conditions (i.e., gastrointestinal tract and endothelial cells) (Baranwal and Mehra, 2017). *MICA mRNA* transcripts are detected in decidual, placental, and trophoblast cells from healthy pregnancies, although the MICA molecule is barely detected on placental tissues (Mincheva-Nilsson et al., 2006; Apps et al., 2008). It has been proposed that soluble MICA (sMICA) in pregnant women may participate in fetal immune escape (Mincheva-Nilsson et al., 2006; Huang et al., 2011), although high levels of sMICA were considered a predictive biomarker for *in vitro* fertilization failure (Porcu-Buisson et al., 2007). Indeed, in pathological situations, *MICA* expression patterns might change. A dimorphism known as MICA-129Val/Met (rs1051792), is reported to influence both sMICA levels and affinity to the NKG2D receptor expressed on cytotoxic cells, including uNK cells. It was observed that soluble NKG2D has a higher affinity to 129Met molecules (range 10- to 50-fold) compared to 129Val MICA (Steinle et al., 2001). Thus, this variant seems relevant in inflammatory disorders (Isernhagen et al., 2016), but its influence on pregnancy disorders is yet to be addressed. Besides, high sMICA levels are observed in PE and other vascular pregnancy disorders, often being absent in healthy pregnancies. Further, sMICA maternal plasma from PE women downregulates NKG2D expression on CD3-CD56+NK cells from healthy donors (Haumonte et al., 2014), suggesting that sMICA impairs vascular remodeling through downregulation of NK effector functions by means of interferon-gamma secretion and cytotoxicity (Haumonte et al., 2014; Zhou et al., 2014). Additionally, microvesicles derived from early placenta harbor MICA which has potential to downregulate NKG2D (Hedlund et al., 2009).

### Non-coding RNAs and Epigenetics

In the era of genomics, next-generation DNA sequencing is becoming a technique accessible to most laboratories. The possibility of massively interrogating millions of DNA strands at the same time has fostered research in the search of causal genetic variation involved in PE pathophysiology (see Yong et al., 2018b). The profile of non-coding RNA (ncRNA) in distinct tissues, body fluids and disorders has revealed a universe of RNAs, which is currently under extensive investigation. Traditionally, ncRNAs are divided into two classes based on size: small ncRNA (< 200 nt) and long ncRNA (>200 nt). The small ncRNA includes microRNA (miRNA), small interfering RNA, small nuclear RNA, small nucleolar RNA, ribosomal RNA, transfer RNA, and P-element-induced-wimpy testis (piwi)-interacting RNA. Their regulatory activity extends to different levels of transcription and post-transcriptional control (Anfossi et al., 2018). Although little is known about long non-coding RNA (lncRNA) functions, they participate in several biological processes such as epigenetic regulation, transcriptional and post-transcriptional control, regulation of miRNAs (by acting as sequence decoys) and acting as scaffolds for protein complex (Li et al., 2014), suggesting a more extensive biological versatility compared to small ncRNAs.

Currently, ncRNAs are considered promising diagnostic tools and disease progression biomarkers in the clinical setting, because their level of presence is expected to correlate with repressive activity (Bounds et al., 2017). In PE, an increasing number of studies have identified potential regulatory ncRNAs, most of them miRNAs such as miR-155 and miR-210 (Bounds et al., 2017; Wei et al., 2017; Winger et al., 2018; Yoffe et al., 2018). miRNAs are of particular interest due to their high stability in body fluids (Brase et al., 2010) and their potential to be released inside microvesicles (Salomon et al., 2017). An emerging role of genetic variants within miRNAs (even virally encoded miRNAs) highlights their influence in the susceptibility to viral infections (Ellwanger et al., 2018).

Despite the promising applications of ncRNAs as biomarkers in distinct pathologies, the increasing complexity for use due to ncRNA heterogeneity as well as the diversity of methodologies implemented for their isolation (Anfossi et al., 2018) highlight some challenges that should be addressed, including the need for sample collection, processing, and analysis standardization in order to increase the feasibility and replicability of studies.

It is clear that epigenetics is a mechanism involved in the development of a healthy pregnancy. Partially methylated domains (PMDs) are regions showing reduced average methylation levels which cover up to 40% of the genome. PMDs are observed in only a few cell types: cultured cells, malignant cells and placental cells (Hansen et al., 2011; Schroeder and LaSalle, 2013). Interestingly, PMD covers 37% of the placental genome, and most of the genes in PMD are repressed, suggesting that repression of specific genes within PMD during pregnancy is needed for healthy development (Schroeder et al., 2013). Following this reasoning, disruption in the epigenetic program could lead to placental dysfunction and associated disorders (Robinson and Price, 2015). Some studies have evaluated the methylation status, or methylome, of the placenta in pregnancy disorders such as preterm birth (Hong et al., 2017), intrauterine growth restriction (Hillman et al., 2015), and PE (Blair et al., 2013; Anton et al., 2014; Chu et al., 2014; Liu et al., 2014). Owing to the fact that different methodologies exist, the comparison between studies is not always possible. Nonetheless, two genes (*DAPK3* and *PAPPA2*) were observed to share methylation patterns in preeclamptic placenta (Blair et al., 2013; Chu et al., 2014; Bianco-Miotto et al., 2016). Apart from pregnancy disorders, the methylation patterns in placental PMD suggest a causal link to autism spectrum disorders because behavioral genes are overrepresented in placental PMD (Schroeder et al., 2016). There is also a suggestion of an interaction between environmental factors and DNA, altering epigenetic features and therefore susceptibility to many disorders including PE (Chelbi and Vaiman, 2008).

Analysis of DNA methylation in cord blood cells may improve our knowledge of epigenetic signatures in pregnancy (and PE) and improve understanding of their implications for adult life. For example, hypomethylation of the 11β-hydroxysteroid dehydrogenase type-2 (*HSD11B2*) gene promoter is suggested to increase fetal glucocorticoid levels identified as risk factors for metabolic diseases (Hu et al., 2014). In a study evaluating cord blood in early preeclamptic women, different sets of genes from lipid metabolism, cellular proliferation and inflammation showed variable levels of methylation in their promoter regions, suggesting that early epigenetic signatures are detected in newborns and could be associated with predisposition to cardiovascular diseases in adulthood (Ching et al., 2015). Nevertheless, whether early risk epigenetic modifications remain constant and act as disease triggers or risk-modifying factors is still an open question.

Genomic imprinting is closely associated with parental origin, which highlights that epigenetic disruption can result in abnormal expression of imprinted genes in the placenta and contributes to PE development. The distal-less homeobox-5 (*DLX5*) gene is paternally imprinted (maternally expressed gene) in normal healthy placenta, but its status is upregulated in PE as a result of the loss of paternal imprinting. *DLX5* was upregulated in up to 70% of PE placentas correlating positively with classical PE markers (i.e., PlGF:sFLT). Of note, overexpression of DLX5 *in vitro* led to reduced proliferation and endoplasmic reticulum stress of trophoblast cells (Zadora et al., 2017). GATA-binding protein 3 (*GATA3*), a gene relevant to trophoblast invasion, was also identified as a candidate for future research concerning dysregulated imprint and pregnancy disorders (Chiu and Chen, 2016; Zadora et al., 2017).

Overall, the methylome opens new perspectives for comprehension of the phenomenon of inherited traits unrelated to classical nucleotide sequence changes in the genome (SNPs or mutations) and how they affect phenotype. The future is promising, but some important issues should be addressed. For example, PMD is overrepresented in the placental methylome, but most of the studies published so far have ignored them, raising the question of whether PMD occurs in specific trophoblast cell lineages or at specific stages of development (Schroeder et al., 2013; Bianco-Miotto et al., 2016). Interestingly, methylation patterns in early extraembryonic tissues resemble those commonly observed in cancer (Smith et al., 2017), implying that comprehension of the epigenomic landscape of these two phenomena would provide some clues to the inherent process of cellular invasion, proliferation, and vasculogenesis. Also, the paradox of high methylation of CpG islands in genes within placental PMD is yet to be addressed. Lastly, future studies should differentiate hypomethylation patterns occurring in PMD regions from those occurring in other genomic regions (Schroeder et al., 2013).

### Placental Microbiome—Friend or Foe

The fact that microorganisms are detected in the placenta, the womb, and the fetus, once thought of as sterile entities, has attracted much attention. The detection of bacterial DNA in the placenta (Aagaard et al., 2014; Collado et al., 2016) and in the amniotic fluid (Collado et al., 2016) has brought the “placental microbiome” into the spotlight. This concept challenges the traditional belief that newborns acquire their first bacteria only as they pass through the birth canal. The observation that *Enterococcus faecium* from human breast milk orally inoculated in pregnant mice can be detected in the amniotic fluid, and the pup's meconium (Jiménez et al., 2005, 2008; Aagaard et al., 2014) further supports the concept of the placental microbiome. In this same line, it seems that the newborn gut microbiome shares similarities to the maternal oral microbiome (Aagaard et al., 2014). The nature of symbiosis between extraembryonic tissues and the local community of microorganisms is still unknown. Although studies support the existence of fetal microbiomes, there is currently skepticism surround the concept, as discussed in other studies (Lauder et al., 2016; Perez-Muñoz et al., 2017).

The presence of placental microbiota in normal pregnancy (Aagaard et al., 2014; Parnell et al., 2017) intuitively implies that an altered microbiome would underlie pregnancy disorders such as chorioamnionitis and preterm birth (Antony et al., 2015; Prince et al., 2016). In this sense, a novel mechanism by which viruses may alter immunologic tolerance to intrauterine bacteria was suggested (Cross et al., 2017). It demonstrated that polymicrobial exposure of human fetal membranes (FM; amnion and chorion) explanted to bacterial LPS and virus [Herpes simplex virus type 2 (HSV2)] samples result in the aberrant expression of IL-1β, which is commonly observed in chorioamnionitis and preterm birth (Gomez-Lopez et al., 2017). The mechanism is not fully understood, however, it involves downregulation of the MER tyrosine kinase proto-oncogene (MERKT) receptor, allowing the activation of Nod-like receptor protein-3 (NLRP3) also known as the NLRP3 inflammasome through a synergistic signaling by LPS/TLR4 (TLR: toll-like receptor-4) and viral double strand dsRNA/TLR3 (dsRNA: double strand RNA) (Cross et al., 2017). It is worth mentioning that some viruses exploit TAM receptors for cell attachment and entry, but whether they are surrogates capable of suppressing TLR signaling is unclear (Best, 2013; Bhattacharyya et al., 2013). This observation is relevant since NLRP3 expression seems to be higher in the placental villi of preeclamptic women compared to normotensive women (Weel et al., 2017). However, if polymicrobial exposure underlies NLRP3 expression in preeclamptic placentas, and if different herpesviruses (i.e., congenital Cytomegalovirus infection) besides the ones evaluated are also able to reduce LPS threshold response are still open questions.

## General Concluding Remarks

In Latin America, several studies approached the molecular basis of PE pathogenesis, documented by the increasing amount of scientific study and its impact on local and international scientific communities. In this endeavor, Brazil and Mexico are at the forefront of scientific production. However, we call attention to the need for studies in other Latin American countries, since these regions are characterized by a highly genetically diverse human population. Additionally, PE and gestational hypertensive disorders are a heavy burden in Latin America, strongly affecting maternal and fetal health.

**Figure 3:**
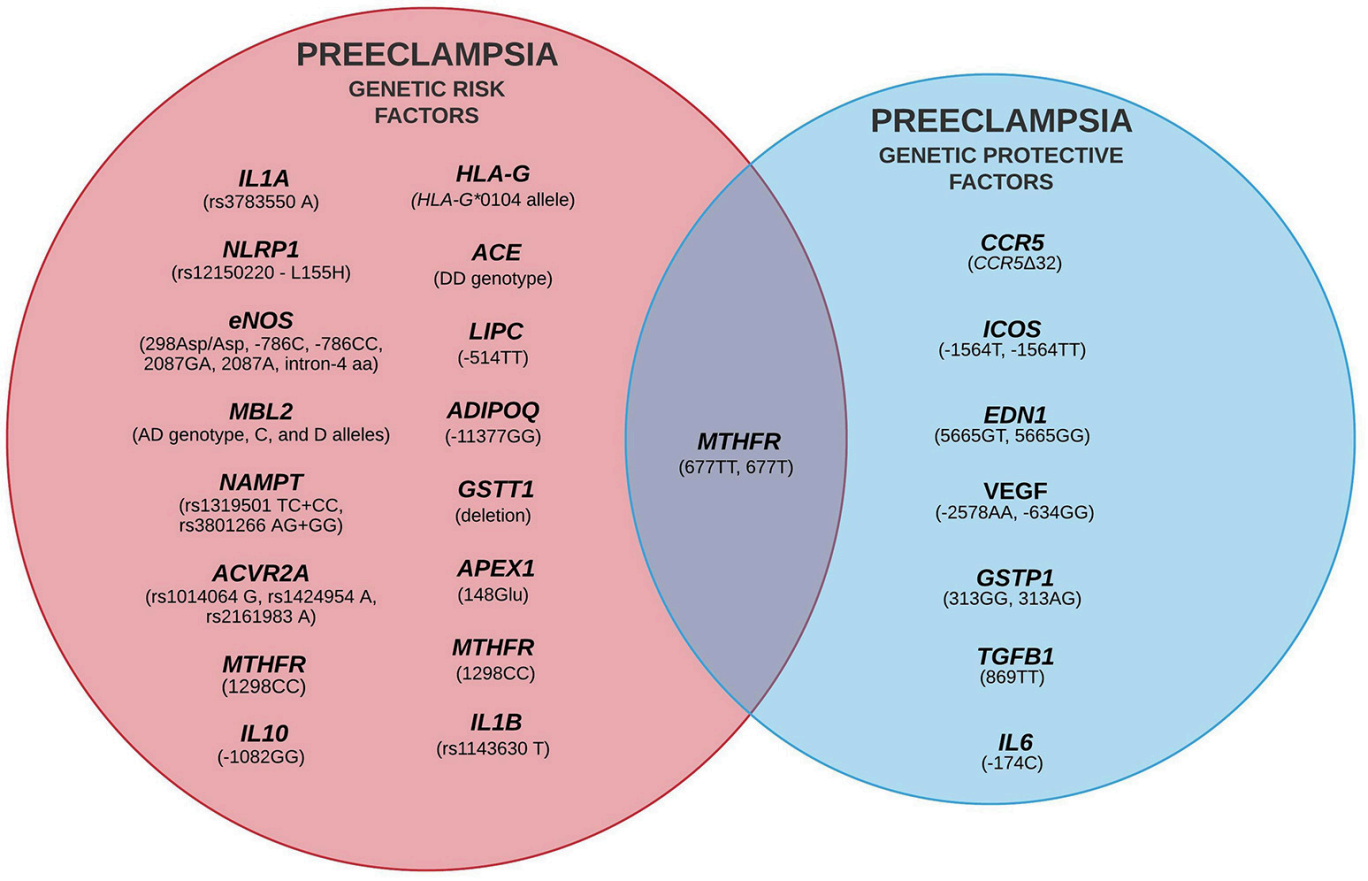
Summary of preeclampsia-associated genetic variants addressed in Latin American studies. Only single locus polymorphisms are shown. Risk and protection associated variants are shown where the intersection represents variants found in both protective and risk factors in different studies. Additional information and haplotypes are detailed in Tables 1–5.

Several genetic variants influencing PE predisposition were reported (Figure 3), some consistently associated with PE across different populations, despite disparities in the genetic/ethnic background inherent in Latin American populations. Genetic intra- and inter-variation have a great influence on genetic predisposition to PE. Although a comprehensive literature review was performed in this study, it may not be representative of the genetic variability present in Latin America since human population studies focus on small samples and therefore may not represent the genetic variability of entire local populations. Additionally, in developing countries, medical specialties (i.e., high-risk pregnancy care) are often centralized in the biggest cities. Therefore, replication of studies in different populations and multicentric collaborative studies are encouraged and would provide a better evaluation of the maternal genetic components of PE development in Latin America. Finally, PE and other hypertensive pregnancy disorders are the primary cause of maternal-fetal morbidity and mortality in low- and middle-income countries, representing a significant burden on public healthcare services. Therefore, it is imperative that public health policies assure prenatal care, perinatal monitoring, and health education in order to reduce the risk of pregnancy-related complications.

## Author Contributions

RM designed the review, planned the topics, wrote the review, reviewed the literature, and designed the figures and tables. VK wrote the review, reviewed the literature, designed the figures, and proofread the review. JC designed the review, contributed to writing the topics and critically reviewed the manuscript.

### Conflict of Interest Statement

The authors declare that the research was conducted in the absence of any commercial or financial relationships that could be construed as a potential conflict of interest.
